# Neo-Adjuvant Treatment in Primary Resectable Pancreatic Cancer: A Systematic Review and PRISMA-Compliant Updated Metanalysis of Oncological Outcomes

**DOI:** 10.3390/cancers15184627

**Published:** 2023-09-19

**Authors:** Raffaello Roesel, Letizia Deantonio, Lorenzo Bernardi, Maria Luisa Garo, Pietro Majno-Hurst, Alberto Vannelli, Marco Cefalì, Maria Celeste Palmarocchi, Maria Carla Valli, Guido Pesola, Alessandra Cristaudi, Sara De Dosso

**Affiliations:** 1 Department of Visceral Surgery, Hospital of Lugano (EOC), 6900 Lugano, Switzerlandpietro.majno-hurst@eoc.ch (P.M.-H.); alessandra.cristaudi@eoc.ch (A.C.); 2 Radiation Oncology Department, Oncology Institute of Southern Switzerland (IOSI), EOC, 6500 Bellinzona, Switzerland; letizia.deantonio@eoc.ch (L.D.); mariacarla.valli@eoc.ch (M.C.V.); 3 Faculty of Biomedical Sciences, Università della Svizzera Italiana (USI), 6900 Lugano, Switzerland; 4 Independent Researcher, 00199 Rome, Italy; marilu.garo@gmail.com; 5 Department of General Surgery, Ospedale Valduce, 22100 Como, Italy; avannelli@valduce.it; 6 Medical Oncology Department, Oncology Institute of Southern Switzerland (IOSI), EOC, 6500 Bellinzona, Switzerland; marco.cefali@eoc.ch (M.C.); mariaceleste.palmarocchi@eoc.ch (M.C.P.); guido.pesola@eoc.ch (G.P.)

**Keywords:** resectable pancreatic cancer, pancreatic adenocarcinoma, neoadjuvant chemotherapy, neoadjuvant chemoradiotherapy, neo-adjuvant treatment

## Abstract

**Simple Summary:**

Pancreatic adenocarcinoma is the fourth leading cause of cancer-related death in industrialized countries. In locally advanced and borderline resectable pancreatic cancer, neoadjuvant therapy (NAT) has been shown to be effective in eliminating potentially circulating tumor cells and distant micrometastases, shrinking local tumors, and identifying high-grade malignancies that do not benefit from surgery. However, in patients with resectable pancreatic adenocarcinoma, who represent 20% of new diagnoses and for whom US followed by adjuvant chemotherapy is the standard of care, NAT is controversial because it carries several potential drawbacks that may prevent surgery and increase the risk of clinical deterioration. Randomized clinical trials, retrospective studies, and a few systematic reviews and meta-analyses reported controversial results, and although the safety and feasibility of such an approach are supported, a wider implementation is still a matter of debate. Considering the different methodological approaches (RCTs vs. retrospective studies), the difficulty in providing high-quality evidence due to small patient numbers, and the emergence of new evidence, an update of the current evidence seems essential to help clinicians and researchers understand the role of NAT and offer a new potentially beneficial treatment approach. Thus, the aim of this systematic review and meta-analysis is to evaluate the role of NAT in prolonging overall survival and disease-free survival and improving R0 and N0 rates compared with upfront resection in patients with resectable pancreatic cancer.

**Abstract:**

Background: Despite advances in treatment, the prognosis of resectable pancreatic adenocarcinoma remains poor. Neoadjuvant therapy (NAT) has gained great interest in hopes of improving survival. However, the results of available studies based on different treatment approaches, such as chemotherapy and chemoradiotherapy, showed contrasting results. The aim of this systematic review and meta-analysis is to clarify the benefit of NAT compared to upfront surgery (US) in primarily resectable pancreatic adenocarcinoma. Methods: A PRISMA literature review identified 139 studies, of which 15 were finally included in the systematic review and meta-analysis. All data from eligible articles was summarized in a systematic summary and then used for the meta-analysis. Specifically, we used HR for OS and DFS and risk estimates (odds ratios) for the R0 resection rate and the N+ rate. The risk of bias was correctly assessed according to the nature of the studies included. Results: From the pooled HRs, OS for NAT patients was better, with an HR for death of 0.80 (95% CI: 0.72–0.90) at a significance level of less than 1%. In the sub-group analysis, no difference was found between patients treated with chemoradiotherapy or chemotherapy exclusively. The meta-analysis of seven studies that reported DFS for NAT resulted in a pooled HR for progression of 0.66 (95% CI: 0.56–0.79) with a significance level of less than 1%. A significantly lower risk of positive lymph nodes (OR: 0.45; 95% CI: 0.32–0.63) and an improved R0 resection rate (OR: 1.70; 95% CI: 1.23–2.36) were also found in patients treated with NAT, despite high heterogeneity. Conclusions: NAT is associated with improved survival for patients with resectable pancreatic adenocarcinoma; however, the optimal treatment strategy has yet to be defined, and further studies are required.

## 1. Introduction

Globally, in 2020, pancreatic adenocarcinoma will be the 14th most common malignancy, with 495,773 new worldwide cases [1] and an average lifetime cumulative risk of occurrence of 1 in 64 [2]. By 2030, pancreatic cancer is expected to become the second cause of cancer-related death, despite survival improvements with current treatments [3]. At the time of diagnosis, pancreatic cancer is considered primarily resectable in 10–20% of cases based on the absence of metastases and loco-regional arterial infiltration [4]. Tumor contact with either the portal or superior mesenteric vein of less than 180° without deformation of the vessel is not considered a contraindication to surgery [5]. In this context, a radical (R0) surgery with standard D2 lymphadenectomy is the cornerstone of treatment [6]. The introduction of adjuvant chemotherapy has improved prognosis and is one of the factors mainly associated with long-term survival [7]. In this regard, FOLFIRINOX is currently the best adjuvant treatment regimen, according to the results of the PRODIGE24 trial, which showed a significantly better median overall survival (OS) of 54 months with FOLFIRINOX compared to 37 months with gemcitabine in a highly selected population [8]. As an alternative, gemcitabine-based regimens can be considered in less fit patients, according to the positive results of the ESPAC-4 trial [9], and gemcitabine alone should be used only in frail patients [10,11]. Nonetheless, only patients with a good performance status and a good recovery after surgery can receive adjuvant chemotherapy, corresponding to half of the overall population of patients with resectable pancreatic cancer. Thus, a considerable proportion of patients are unable to start adjuvant treatment due to the morbidity of pancreatic surgery, and an even greater proportion of resected patients fail to complete all planned cycles of adjuvant therapy [12,13,14].

Despite the current available treatments, the 5-year OS rate is still poor, close to 20% [15,16], with a 3-year disease-free survival (DFS) rate between 24% and 39% [10]. Therefore, the development of new strategies to improve clinical results in this setting is currently a priority. In this regard, considerable interest has been focused on the role of neoadjuvant therapy (NAT), which has been demonstrated to improve OS without necessarily increasing the resection rate in patients with borderline resectable tumors [17,18]. The potential advantages of a neoadjuvant strategy are the eradication of micro-metastases and a higher chemotherapy completion rate compared to the adjuvant setting. Furthermore, NAT may increase the microscopically margin-negative resection rate (R0 rate), nodal negative disease (N0), and may select patients with rapidly progressive tumors, thus sparing ineffective surgery. On the other hand, NAT-related adverse events could worsen the patient’s performance status, thereby delaying surgery. These potential conflicts can only be clarified by evidence from randomized trials comparing neoadjuvant and adjuvant treatment strategies. Currently, in the setting of resectable pancreatic adenocarcinoma, there are only a few clinical studies comparing NAT versus upfront surgery (US), reporting controversial results. The aim of the present systematic review and meta-analysis is to evaluate the role of NAT in prolonging OS and DFS and improving R0 and N0 rates compared with upfront resection.

## 2. Materials and Methods

This work was conducted in accordance with PRISMA guidelines [19] and registered on PROSPERO (CRD42022382272). The components of the PICO questions were: (population) patients with resectable pancreatic cancer; (intervention) neoadjuvant treatment (NAT); (comparator) upfront surgery; (outcome): overall survival, disease-free survival, R0 resection rate, and positive lymph node rate.

### 2.1. Eligibility Criteria

Peer-reviewed research articles were considered. Eligible studies were selected according to the following criteria: (1) RCTs, prospective or retrospective studies comparing the effects of NAT vs. US; (2) studies including resectable patients with pancreatic cancer; (3) studies reporting primarily overall survival (OS) and disease-free survival (DFS); (4) studies using chemotherapy or chemoradiotherapy as a preoperative neoadjuvant strategy. We considered only papers where the definition of resectability was reported and based on: (1) no extra-pancreatic disease; (2) no tumor extension to the superior mesenteric artery or celiac axis; and (3) limited (<180°) and/or no occlusion of the superior mesenteric vein (SMV) or the SMV-portal vein (PV). Furthermore, any definition of residual disease (R-status) after surgery was accepted.

Studies with insufficient information, overlapping samples, reviews, meta-analyses, studies without complete data from the resectable disease cohort, or studies that included only preoperative radiotherapy as a neoadjuvant strategy were excluded. For overlapping samples, we used the most recent publication or the one with complete information; for studies, one of which was an update of the previous ones, we used the most updated information.

### 2.2. Search Strategy

A systematic search strategy was carried out on PubMed, EMBASE, and the Cochrane Register of Controlled Trials, irrespective of language or publication date, from November 2022 to February 2023.

The search strategy included the following terms: (“pancreas” OR “pancreatic”) AND (“cancer” OR “carcinoma” OR “adenocarcinoma” OR “tumour” OR “neoplasm”) AND (“chemotherapy” OR “irradiation” OR “radiotherapy” OR “radiation therapy” OR “chemoradiotherapy”) AND (“surgery” OR “resectable” OR “up-front resectable”) AND (“neoadjuvant” OR “preoperative”).

Removal of duplicates and screening of titles/abstracts were carried out by two independent reviewers (RR and LB). The full texts of the remaining potentially relevant articles that met the inclusion and exclusion criteria were retrieved and reviewed by two further independent reviewers (MG and AC). Any disagreement was discussed until a decision was reached by consensus. The final eligibility of each study was checked, and the reasons for exclusion were recorded. Two authors (RR and LB) made the final selection of articles. In cases of disagreement, other authors were consulted to reach consensus.

### 2.3. Data Extraction

Two authors (RR and LB) independently extracted data from the full texts of the studies that met the inclusion criteria. Discrepancies were resolved through team discussion. The authors of the included studies were not contacted to obtain unpublished data. In the case of incomplete information from primary studies, data were extracted from two previous meta-analyses [20,21].

The data collected included:

1. Study characteristics: first author, year, country, observation period, and study design.
2. Sample size: total, NAT, and US sample size, respectively.
3. Patients’ demographic characteristics: gender and age.
4. Primary outcome: survival data (OS and DFS).
5. Secondary outcome: R0 resection rate and positive lymph node rate (N+).

For the primary outcomes, we obtained hazard ratio estimates (HR) and 95% CI directly from the individual studies if they were provided by the authors. Otherwise, survival data were extracted from Kaplan–Meier curves with Digitizelt software 2.5.10 (DigitizeIt, Braunschweig, Germany) and subsequently reported in STATA17 (StataCorp., College Station, TX, USA) to determine the HR estimate and its variants. For RCTs, only intention-to-treat analyses were used.

### 2.4. Risk of Bias—Quality Assessment

The quality of RCTs was independently assessed by two authors using the Cochrane Risk of Bias Tool (RoB2). Five domains of bias (i.e., randomization process, deviations from planned interventions, missing outcome data, outcome measurement, and selection of reported outcomes) were assessed and reported according to the Cochrane Handbook for Systematic Reviews of Interventions [22]. A judgment of "high" indicated a high risk of bias, "low" indicated a low risk of bias, and "some concerns" indicated the presence of bias due to a lack of information or uncertainty about the potential for bias. Studies were thus categorized as having a low or high risk of bias or some concerns.

For non-RCT studies, the risk of bias was assessed using the Newcastle-Ottawa Quality Scale (NOS) [23]. The instrument consists of eight items, four of which relate to selection, one to comparability, and three to exposure. A score of less than 4 points indicates a high risk of bias; a score of 5 indicates a moderate risk of bias; and a score of more than 5 indicates a low risk of bias.

The risk of bias was assessed by three independent authors (RR, LB, and AC). Possible disagreements about the quality score were resolved through discussion and consensus among all authors.

### 2.5. Statistical Analysis

All data from eligible articles were summarized in a systematic summary and then used for the meta-analysis. Specifically, we used HR for OS and DFS and risk estimates (odds ratios) for the R0 resection rate and the N+ rate. A random-effects approach (DerSimonian-Laird) was used, hypothesizing a possible heterogeneity between studies due to differences in study designs, observation periods, patient characteristics, neoadjuvant and adjuvant therapies, and follow-up duration. In addition, heterogeneity was assessed using the Cochrane Q-test and the I² statistic according to the Cochrane Handbook for Systematic Reviews of Interventions [24]. Outliers as a possible source of heterogeneity were investigated using the Galbraith plot for OS and DFS and the L’Abbè plot for R0 resection rate and N+ rate. Using the Galbraith plot, we analyzed which studies fell outside the 95% CI range, while using the L’Abbè plot, we assessed which studies deviated significantly from the effect size line. For the sample size subgroup analysis, we set a cut-off of 150 patients, which was chosen after determining the median of the total sample size of the included studies (167) and rounding up to the nearest multiple of 50.

We also performed a cumulative meta-analysis to track the accumulation of evidence per year. Finally, we performed a “leave-one-out” analysis (one study removed) as a sensitive analysis to examine the impact of individual studies on the overall prevalence results. Publication bias was assessed according to Cochrane guidelines if the total number of studies was ≥10. All analyses were performed using STATA18 (StataCorp., College Station, TX, USA).

## 3. Results

### 3.1. Search Results and Studies Characteristics

**Figure 1:**
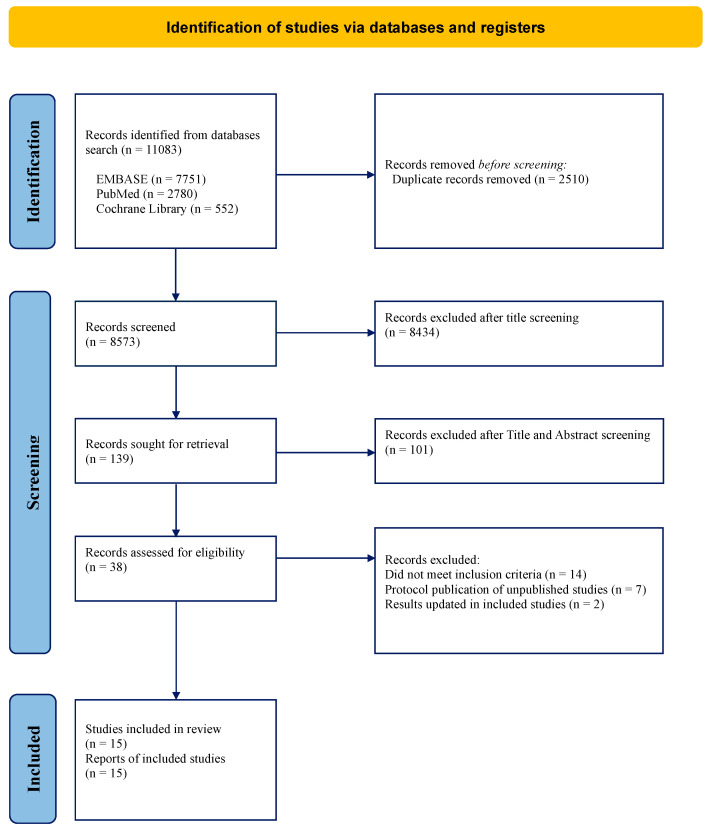
PRISMA Flow-chart.

The search strategy identified 11,083 articles from databases (EMBASE: 7751; PubMed: 2780; Cochrane Library: 522) (Figure 1). After excluding duplicates (n = 2510), 8573 articles were screened by title, of which 8434 were excluded by title and a further 101 by title and abstract. Thirty-eight articles met the inclusion criteria and were subsequently screened in full text. Of these, 23 were excluded (14 did not meet the inclusion criteria, 7 were found to be protocols of unpublished data, and 2 were updated in included studies). Finally, 15 studies from databases were included in this work [17,25,26,27,28,29,30,31,32,33,34,35,36,37,38]. In the study by Birrier et al., we considered only the results from their RCT, excluding those from Casadei et al. and Golcher et al., whereas the results from Casadei et al. and Golcher et al. were retrieved from the original publication [27,28,30].

**Figure 2:**
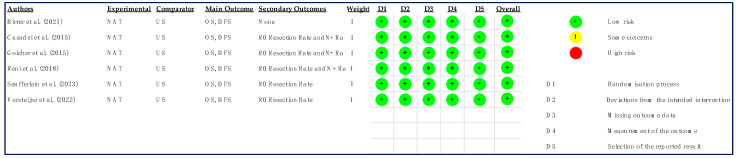
Risk of Bias—RCT [18,26,27,29,32,34].

The quality assessment did not reveal any specific concerns in terms of bias (Figure 2 and Table 1).

### 3.2. Studies Characteristics

Sixteen thousand seven hundred and thirty-one (16,731) patients with a mean male-to-female ratio of 1.32 were included (Table 2 and Supplementary Materials Table S1), of whom 4166 underwent NAT and 12,565 US were included in this meta-analysis. Seven studies were retrospective studies, six were RCTs, and in two, the study design was not reported. Five studies were conducted in the USA [30,31,33,36,37], two in Italy [27,32], Japan [28,35] and Germany [29,34] respectively, and one each in France [25], Switzerland [26], the Netherlands [18] and Korea [38]. The longest study—a retrospective study—examined an observation period of 32 years from 1989 to 2021 [37], while the shortest—one RCT—had a follow-up of 4 years from 2015 to 2019 [34]. Seven studies had a total sample size of more than 150 patients, with two studies including 6802 [37] and 8020 [30] patients, respectively. The median OS for NAT ranged from 15 months [25] to 50.2 months [35], while for the US it ranged from 14.3 months [18] to 32.7 months [35]. Almost all studies used gemcitabine as neoadjuvant therapy (as monotherapy or in combination with other drugs) and as adjuvant chemotherapy after surgery; nine studies used a combination of chemotherapy and radiotherapy as neoadjuvant therapy. More active regimens with modern chemotherapy combinations (PEXG: cisplatin, epirubicin, gemcitabine and capecitabine; Gemcitabine plus Nab-paclitaxel; FOLFIRINOX) were used only in three recent studies [32,34,38].

### 3.3. Overall Survival

**Figure 3:**
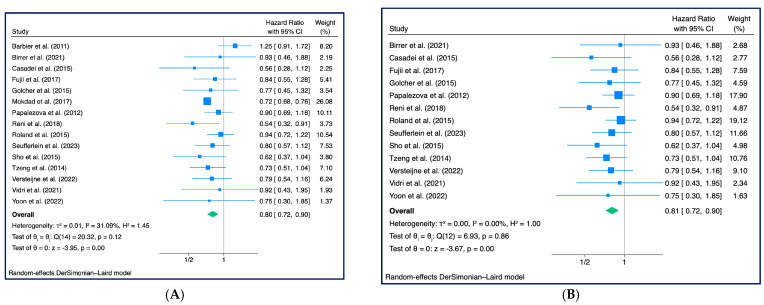
Overall Survival: (**A**) Forest Plot [18,25,26,27,28,29,30,31,32,33,34,35,36,37,38]; (**B**) Overall Survival without outliers [18,26,27,28,29,31,32,33,34,35,36,37,38] (n = 13) (outlier: Barbier et al. 2011 [25], Mokdad et al. 2017 [30]).

All fifteen studies with a total of 16,371 patients were included to investigate the role of NAT on OS. From the pooled HRs, OS for NAT patients was better, with an HR for death of 0.80 (95% CI: 0.72–0.90) with a significance level of less than 1% (z = −3.95, *p* < 0.001, *τ*2 = 0.01, I2 = 31.09%) (Figure 3A). Two studies [25,30] were detected outside the shaded area in the Galbraith plot (Supplementary Materials: Figure S1). The pooled HR excluding this outlier was 0.81 (95% CI: 0.72–0.90; z = −3.67, *p* < 0.001, *τ*2 = 0.00, I2 = 0.00%) (Figure 3B).

**Figure 4:**
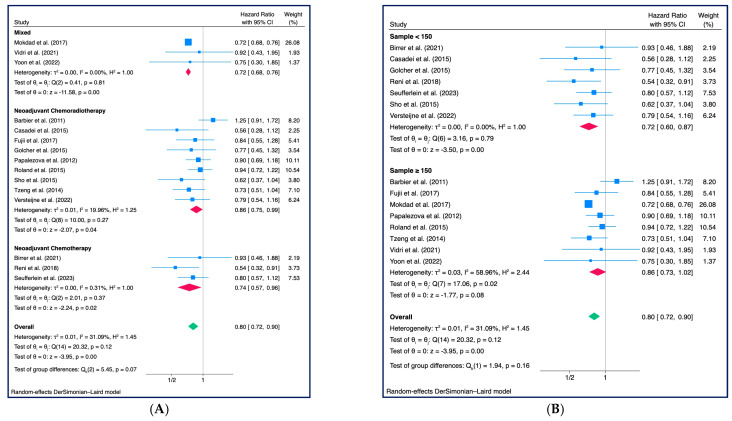
Overall Survival: (**A**) Subgroup analysis related to the use of chemotherapy vs. chemoradiotherapy—Mixed refers to 3 studies that included patients treated both with and without radiotherapy; (**B**) Subgroup analysis related to sample size (<150 pz. vs. ≥150 pz.) [18,25,26,27,28,29,30,31,32,33,34,35,36,37,38].

For studies that included patients treated with chemoradiotherapy as neoadjuvant treatment, the HR for death was 0.86 (95% CI: 0.75–0.99, z = −2.07, *p* = 0.04, *τ*2 = 0.01, I2 = 19.96%), similar to that observed from studies that included only patients treated with neoadjuvant chemotherapy (HR: 0.74, 95% CI: 0.57–0.96, z = −2.24, *p* = 0.02, *τ*2 = 0.00, I2 = 0.31%). HR resulted slightly lower for studies that included patients who received either neoadjuvant chemotherapy or chemoradiotherapy (HR: 0.72, 95% CI: 0.68–0.76, z = −11.58, *p* < 0.001, *τ*2 = 0.00, I2 = 0.00%) (Figure 4A). A significantly lower HR was observed in studies that included fewer than 150 patients (HR: 0.72, 95% CI: 0.60–0.87, z = −3.50, *p* < 0.001, *τ*2 = 0.01, I2 = 0.00%), while studies that included more than 150 patients showed a non-significantly lower HR and a moderate degree of heterogeneity (HR: 0.86, 95% CI: 0.73–1.02, z = −1.77, *p* = 0.08, *τ*2 = 0.03, I2 = 58.96%) (Figure 4B).

The overall trend for HR was confirmed in the subgroup analysis of RCTs, from which HR for death was 0.74 (95% CI: 0.61–0.90, z = −3.06, *p* < 0.001, *τ*2 = 0.01, I2 = 0.00%), while no significant HR emerged from retrospective study analysis (HR: 0.90, 95% CI: 0.76–1.06, z = −1.24, *p* = 0.21, *τ*2 = 0.01, I2 = 21.98%) (Supplementary Materials—Figure S2).

The cumulative analysis revealed a clear trend from 2011 to 2022, showing how HR stabilized at 0.80 (95% CI: 0.72–0.91) (Supplementary Materials—Figure S3). In the leave-one-out analysis, one study deviated significantly from the overall effect [25], as already shown in the Galbraith plot (Supplementary Materials—Figure S4). There was no particular publication bias (Supplementary Materials—Figure S5), which was also confirmed by Egger’s test for small study effects (*p* = 0.698).

### 3.4. Disease-Free Survival

**Figure 5:**
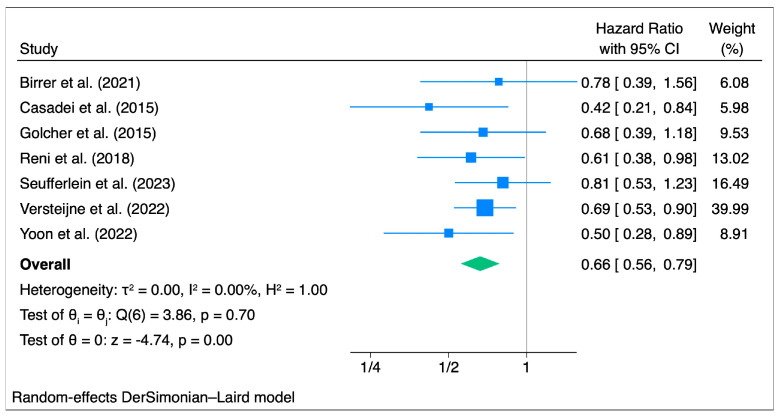
Disease Free Survival (n = 7): Forest Plot [18,26,27,29,32,34,38].

Seven studies with a total of 653 patients were included in the pooled HR for DFS. From the pooled HRs, the DFS for NAT was better, with an HFR for progression of 0.66 (95% CI: 0.56–0.79) and a significance level of less than 1% (z = −4.74, *p* < 0.001, *τ*2 = 0.00, I2 = 0.00%) (Figure 5). The Galbraith plot showed no outliers (Supplementary Materials—Figure S6).

Subgroup analysis showed that patients treated with neoadjuvant chemoradiotherapy had a HR for progression of 0.65 (95% CI: 0.52, 0.82, z = −3.65, *p* < 0.001, *τ*2 = 0.00, I2 = 0.00%), which was slightly lower than that for patients treated with neoadjuvant chemotherapy (HR: 0.73, 95% CI: 0.54–0.97, z = −2.20, *p* = 0.03, *τ*2 = 0.00, I2 = 0.00%), while no conclusions could be drawn for the studies that included patients treated with both neoadjuvant chemotherapy and chemoradiotherapy, as only one study was included (Supplementary Materials—Figure S7). Analysis of only RCTs confirmed the significant HR for progression (HR: 0.68, 95% CI: 0.57–0.81, z = −4.22, *p* < 0.001, *τ*2 = 0.00, I2 = 0.00%) (Supplementary Materials—Figure S8).

In the cumulative analysis, an increase in HR was found over the years (Supplementary Materials—Figure S9), while no significant changes were found in HR from the leave-one-out analysis (Supplementary Materials—Figure S10). Publication bias was not detected because the number of studies was less than 10.

### 3.5. R0 Resection Rate

**Figure 6:**
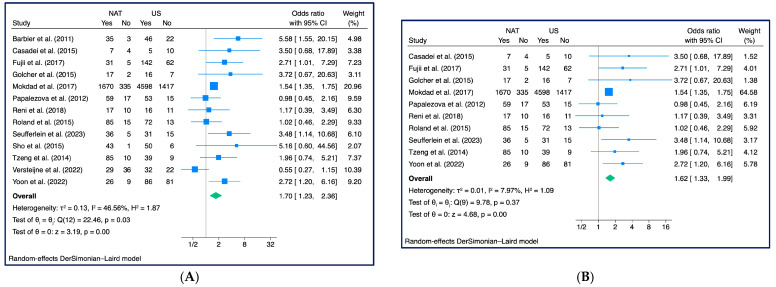
R0 Rate (n = 13): (**A**) Forest Plot [18,25,27,28,29,30,31,32,33,34,35,36,38]; (**B**) Forest Plot without outliers [27,28,29,30,31,32,33,34,36,38] (three outliers: Barbier et al. 2011 [25], Sho et al. 2015 [35], Verstejine et al. 2022 [18])—Note: for Verstejine et al. 2022 [18] data were extracted from the Verstejine et al. 2020 [17].

Thirteen studies with a total of 16,461 patients were included. In the pooled HR, NAT showed a better R0 resection rate than US (OR: 1.70, 95% CI: 1.23–2.36, z = 3.19, *p* < 0.001, *τ*2 = 0.13, I2 = 46.56%) (Figure 6A). After analyzing possible outliers through the L’Abbè plot (Supplementary Materials—Figure S11), the OR recalculated without the three identified outliers showed a slight decrease (OR = 1.62, 95% CI: 1.33–1.99, z = 4.68, *p* < 0.001, *τ*2 = 0.01, I2 = 7.97%), but without losing its significance level (Figure 6B).

Subgroup analysis showed significantly higher HR from retrospective studies (HR: 1.89, 95% CI: 1.09–3.29, z = 2.27, *p* = 0.02, *τ*2 = 0.20, I2 = 44.71%), while such a significant trend was not confirmed pooling results from RCTs (HR: 1.70, 95% CI: 0.70–4.08, z = 1.18, *p* = 0.24, *τ*2 = 0.61, I2 = 63.91%), among which a significant heterogeneity occurred (Supplementary Materials—Figure S12).

No particular trend emerged from the cumulative meta-analysis (Supplementary Materials—Figure S13), and no significant deviation from the effect size was found in the leave-one-out analysis (Supplementary Materials—Figure S14). In the publication bias, a slight asymmetry was observed in the funnel plot (Supplementary Materials—Figure S15), although Egger’s test for small study effects did not reveal a statistically significant indication (*p* = 0.096).

### 3.6. N+ Rate

**Figure 7:**
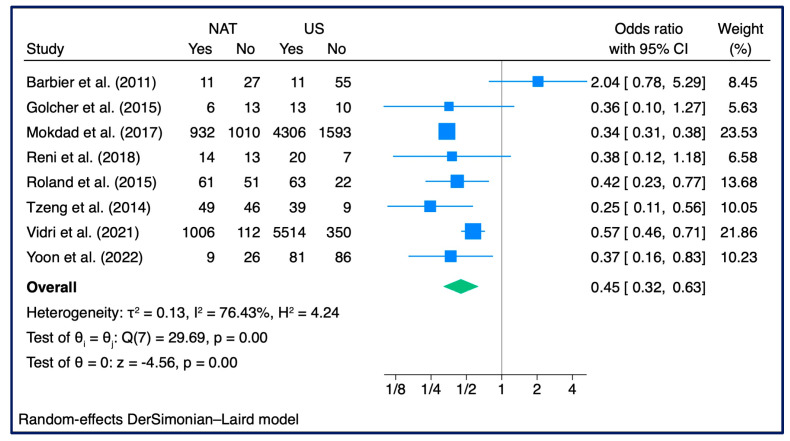
N+ Rate (n = 8): Forest Plot [25,29,30,32,36,37,38].

A meta-analysis of positive lymph nodes performed on a total of eight studies (n = 15,799 patients) showed a lower risk of positive lymph nodes in patients treated with NAT (OR: 0.45, 95% CI: 0.32–0.63, z = −4.56, *τ*2 = 0.13, I2 = 76.43%) (Figure 7). In the quantitative investigation of the possible causes for the large heterogeneity, no outliers occurred (Supplementary Materials—Figure S16), and there was also no significant element of bias in a particular study (sensitive analysis) (Supplementary Materials—Figure S17), while a clear trend towards stabilization of risk over the years emerged (Supplementary Materials—Figure S18).

The overall trend was also confirmed in the subgroup analysis performed, including RCTs (HR: 0.37, 95% CI: 0.16–0.86, z = −2.31, *p* = 0.02, *τ*2 = 0.00, I2 = 0.00%) and retrospective studies (HR: 0.52, 95% CI: 0.32–0.85, z = −2.61, *p* = 0.01, *τ*2 = 0.20, I2 = 68.39%), although in the latter subgroup a moderate level of heterogeneity emerged (Supplementary Materials—Figure S19).

Publication bias was not determined.

## 4. Discussion

Upfront resection with adjuvant chemotherapy still represents the standard of care for patients with resectable pancreatic cancer. Although a highly selected population (i.e., patients younger than 79 years, bilirubin <1.5 ULN, R0 or R1 resection within 12 weeks before randomization, post-surgical CT or MRI, post-operative CA 19-9 < 180 U/mL) was suitable for an intensive regimen (FOLFIRINOX), the prognosis is still poor, with a median OS of up to 53.5 months [8]. In addition, only patients with a good performance status and a good recovery after surgery can be treated with adjuvant chemotherapy, corresponding to half of the overall population of patients with resectable pancreatic cancer. In such a scenario, novel strategies are required to improve the outcome. Neoadjuvant/perioperative treatments have been shown to improve DFS and OS in other gastrointestinal cancers, such as rectal, oesophageal, and gastroesophageal cancer, in which such treatment strategies have long since been established as the standard of care. NAT has gained interest given the systemic nature of pancreatic adenocarcinoma and the difficulty of delivering planned adjuvant treatment to a limited number of patients. In addition, NAT may improve R0 and N0 resections, a well-known prognostic factor, and exclude from futile surgery those patients with rapidly progressive tumors. Since 2019, the National Comprehensive Cancer Network (NCCN) guidelines recommend US followed by adjuvant treatment for resectable pancreatic cancer, but advise considering NAT in those patients with high-risk features (large primary tumors, regional lymph nodes, elevated Ca19.9) [4].

Currently, there are a few clinical studies comparing NAT versus US in resectable pancreatic adenocarcinoma, reporting controversial results. Furthermore, considering the different methodological approaches (RCTs and retrospective studies), the difficulty in providing high-quality evidence due to the small sample size, and the emergence of new evidence, an update of the current evidence seems essential to help clinicians and researchers understand the potential role of NAT. Thus, the aim of this systematic review and meta-analysis was to evaluate the role of NAT for patients with resectable pancreatic cancer in improving OS and DFS and improving R0 and N0 rates compared with upfront resection.

This meta-analysis of 15 studies, including RCTs and retrospective studies published between 2011 and 2023, showed that NAT in resectable pancreatic adenocarcinoma significantly improved OS, DFS, N0, and R0 resection rates. Almost all studies used gemcitabine as neoadjuvant (monotherapy or in combination with other drugs) and adjuvant chemotherapy after surgery; nine studies used a combination of chemotherapy and radiotherapy as NAT. More active regimens with modern chemotherapy combinations were used only in three recent studies included in our analysis [32,34,38]. In 2018, Reni et al. demonstrated promising efficacy with a multiagent combination (PEXG: cisplatin, epirubicin, gemcitabine, and capecitabine) as a neoadjuvant chemotherapy strategy in a small Phase II trial on 88 patients [32]. A median survival of 38.2 months and 3- and 5-year OS of 55% and 49%, respectively, were observed in patients who received neoadjuvant PEXG, meeting the predefined criteria for success. Notably, the results of this trial suggested that the upfront use of a more effective chemotherapy regimen might reduce the risk of progression during the preoperative phase or during the first 3 months of the adjuvant phase. Nonetheless, since the trial began, the standard of care for adjuvant therapy has changed, and other chemotherapy regimens have been developed. Accordingly, the authors decided to interrupt the planned Phase III trial.

In the randomized phase II NEONAX trial, a perioperative or only adjuvant chemotherapy strategy with Gemcitabine plus Nab-Paclitaxel was compared in patients with resectable pancreatic adenocarcinoma. The primary endpoint of DFS > 55% at 18 months, based on data from CONKO-001 [11], was not reached in both arms [34]. However, a major difference was demonstrated in median DFS (5.9 versus 17.9 months) in the adjuvant arm for the intention to treat (ITT) population versus modified ITT (defined excluding those trial participants in the ITT population that did not receive the intended study interventions), due to several patients not being able to start adjuvant treatment for different reasons. This finding may be explained by the difference in chemotherapy exposure, with 90% of patients in the NAT arm completing pre-operative chemotherapy and 58% of patients starting adjuvant chemotherapy in the other arm. Thus, this trial demonstrates that chemotherapy delivery is most likely the most important non-surgical factor in improving the survival of patients with resectable pancreatic cancer.

In a retrospective analysis published in 2022, 202 patients from a single Korean hospital who underwent curative-intent pancreatic surgery for resectable pancreatic adenocarcinoma were divided into two groups: those undergoing US (n = 167, 82.7%) and those receiving neoadjuvant therapy followed by surgery (n = 35, 17.3%) [38]. Among this latest group, chemoradiotherapy was delivered in 43% of cases and chemotherapy alone in 57%. In most cases (n = 17), the regimen of choice was FOLFIRINOX, while Gemcitabine plus Nab-Paclitaxel, CDDP, or Erlotinib were chosen in the remaining 3 patients. As a result, a significantly better DFS was observed with the addition of neoadjuvant therapy to the management of resectable pancreatic adenocarcinoma.

Additionally, the Phase II trial SWOG1505 explored the potential benefit of either mFOLFIRINOX or gemcitabine/nab-paclitaxel as NAT in resectable pancreatic adenocarcinoma [39]. Both perioperative regimens did not show a significant survival benefit over historical data from adjuvant trials; however, a formal comparison should be made with caution. In fact, in adjuvant trials, patients were randomized postoperatively, and therefore only patients with a complete recovery after surgery and who were fit for chemotherapy were considered, whereas in SWOG1505 patients were randomized at the time of diagnosis. On the other hand, encouraging signs of a beneficial role of NAT were highlighted, with a high R0 resection rate (>80% vs. 40–60% in adjuvant trials), high N0 resections (40% vs. 20–30%), high chemotherapy exposure (90%), and a low incidence of grade 3–5 post-operative complications (16%).

Recently, negative results of a randomized phase 2 study were presented at ASCO 2023. The NORPACT-1 trial randomized 140 patients with resectable pancreatic cancer to receive upfront surgery and adjuvant FOLFIRINOX for six months or a short-course neoadjuvant FOLFIRINOX for two months, followed by surgery and the rest of the chemotherapy postoperatively [40]. According to the preliminary results, the upfront surgery group seems to be doing better than the group who received neoadjuvant therapy, with a median OS in the ITT population of 38.5 months (upfront surgery) versus 25.1 months (neoadjuvant) and HR 1.52 (95% CI, 0.94–2.46), *p* = 0.096. In the neoadjuvant cohort, a considerable drop-out of patients was observed, as only 40 of 77 completed the planned preoperative program. Furthermore, a R0 rate of 56% with neoadjuvant therapy and 39% with upfront surgery were found, figures that are unusual by modern surgical standards. Also, the higher R0 and N0 resections in the neoadjuvant arm surprisingly did not translate into a survival advantage. Given all these limitations, the study does not seem to allow for any conclusion

Among the nine studies included in our research analyzing radiotherapy mainly concomitant to gemcitabine-based chemotherapy, three were randomized clinical trials reporting interesting findings deserving some consideration. The phase III randomized PREOPANC trial evaluated a strategy with three cycles of neoadjuvant gemcitabine combined with radiotherapy (36 Gy in 15 fractions) in the second cycle versus US and adjuvant gemcitabine in resectable and borderline resectable pancreatic adenocarcinoma. In the long term, updated results showed that neoadjuvant treatment improved OS in the overall cohort, but in the subgroup of resectable pancreatic adenocarcinoma, OS was not significantly improved (*p* = 0.23). However, the 5-year OS rate showed a clinically relevant improvement of 14%, including resectable pancreatic adenocarcinoma. These data seem to confirm the importance of long-term follow-up to detect a survival difference with a clinical impact. This result was in line, for example, with the CONKO-001 trial, in which survival differences were found after longer follow-up. In this regard, a high rate of progression and death events has been seen in the first year in both the NAT and US groups. We can argue that such a NAT schedule is not able to prevent early progression and that more effective schedules are required. Moreover, this recent trial showed that large RCTs exploring NAT for pancreatic adenocarcinoma can be conducted with satisfactory and rapid accrual. This was in contrast with two previous RCTs by Casadei et al. and Golcher et al. [27,29] that explored gemcitabine-based chemotherapy and conventionally fractionated radiotherapy compared to the US. Both trials experienced poor accrual and were terminated early. In Golcher’s trial, neither the median OS nor the R0 resection rate were significantly different between the two groups. Similarly, in Casadei’s trial, R0 resection and survival rates were not different between the groups of patients. The low power of the studies suggested that the results were most likely due to unpowered data caused by premature study termination. The retrospective study by Sho et al. reported the findings of 100 patients treated with neoadjuvant gemcitabine-based chemotherapy and radiotherapy or US [35]. The study notably showed that median survival time was significantly better in patients who completed adjuvant chemotherapy than in those not able to complete the treatment. This data pointed out the significant impact of adjuvant chemotherapy. The importance of the overall duration of systemic treatment was also shown by Reni et al. [32], and despite the not significantly different results between NAT and US because of poor accrual, Golcher et al. also demonstrated higher multimodality therapy completion in the NAT group (58% versus 30%) [29]. Similarly, Versteijne et al. stated that the total cumulative dose of chemotherapy was significantly higher in the neoadjuvant chemoradiotherapy group [18], supporting the hypothesis of better tolerability with neoadjuvant administration. The matched cohort series by Mokdad et al. [30] further supported that there was a significant dropout for patients after pancreatic resection that precludes completion of adjuvant therapy, which is known to increase survival in this biologically aggressive disease.

Interestingly, in our analysis, a clear trend for OS in favor of NAT from 2011 to 2023 has been demonstrated, showing how the hazard ratio stabilized at 0.80 (95% CI: 0.72–0.91). A possible explanation can be found in the improvement in chemotherapy protocols, with the above-mentioned modern and more active multiagent regimens; in the prospective and randomized nature of more recent studies with well-defined inclusion criteria; and maybe in the better quality of the radiotherapy schedule; however, a quality assurance procedure was performed only in the Versteijne trial, and therefore it is difficult to derive stronger conclusions.

The present meta-analysis demonstrated that the R0 resection rate was higher in NAT than in the US. The increasing rate of free margin resection is an important prognostic factor, reducing the risk of local recurrence and having a positive impact on survival rates. Moreover, all studies stated that neoadjuvant treatment allows a better selection of patients that do have an advantage from surgery and prevents patients with rapid disease progression from having an unnecessary major intra-abdominal operation.

Although our meta-analysis included both randomized clinical trials and retrospective studies comparing neoadjuvant strategies (e.g., chemotherapy and chemoradiotherapy) versus US in a setting of resectable pancreatic adenocarcinoma, OS, DFS, and N+ rates were also confirmed by the subgroup analysis, which included only RCTs. Our positive results in favor of NAT are in contrast with a recent meta-analysis in which neoadjuvant chemotherapy or chemoradiation did not improve either DFS or OS compared to US followed by adjuvant treatment [40]. This discrepancy can be explained mainly by three reasons: First, our study followed a different logical-statistical approach. Second, in the case of incomplete results, we determined the hazard ratio using the methodological approach described in the “materials and methods” section. Third, due to different eligibility criteria, two RCT studies published only as proceeding abstracts [41,42] were included in Uson Junior were not included in our study.

Our results provide evidence in favor of NAT in patients with resectable pancreatic adenocarcinoma. The promising results and feasibility of the PREOPANC trial and the established advantage of multiagent chemotherapy schedules, such as FOLFIRINOX, have led to the conduct of several ongoing trials in the setting of resectable pancreatic adenocarcinoma: the PREOPANC-2 trial is comparing neoadjuvant FOLFIRINOX with neoadjuvant gemcitabine-based chemoradiotherapy [43], and two randomized trials are investigating neoadjuvant FOLFIRINOX (ALLIANCE A021806 [clinicaltrial.govNCT04340141], PREOPANC-3 [clinicaltrial.gov NCT04927780]). The definitive results from these trials will further clarify whether or not NAT is supported in resectable pancreatic cancers.

However, some limitations should be addressed. Most studies were designed and enrolled patients, or were retrospectively retrieved, when gemcitabine represented the standard of care. This monotherapy regimen is nowadays considered obsolete and used in fragile patients. Only three studies included in our analysis used more active chemotherapy regimens. In addition, among trials with a chemoradiotherapy neoadjuvant schedule, poor details were reported on radiotherapy technique and treatment volume definition, in particular concerning prophylactic irradiation or omission of locoregional negative lymph nodes, which is an important factor for N0 rates. Furthermore, some studies were prematurely closed due to poor accrual. Lastly, the published studies did not stratify patients considering potential prognostic biomarkers such as circulating tumor DNA (ctDNA), which is recently gaining interest in pancreatic cancer [44].

Despite these limitations, the reported advantage of NAT could lead to a change from the traditional approach for resectable pancreatic cancer with US, and NAT may be part of the therapeutic algorithm during multidisciplinary discussion with the chance of therapeutic benefit and eventual cure.

## 5. Conclusions and Future Directions

The present meta-analysis evaluating the survival benefit of NAT sustains its implementation in the context of primarily resectable pancreatic adenocarcinoma. However, standardization of therapy regimen, duration, and amount of therapy, along with the integration of radiation therapy, require further evaluation. The ongoing trials may answer these questions. As future perspectives, molecular or radiomic biomarkers, as well as liquid biopsy based on ctDNA analysis and other molecular biomarkers, may help to predict recurrence and survival and could guide the selection of patients who most benefit from a certain treatment or therapeutic strategy according to response assessment before surgery.

## Figures and Tables

**Table 1:** Risk of Bias—Newcastle-Ottawa scale assessment (NOS).

Author	Is the Case Definition Adequate?	Representativeness of the Cases	Selection of Controls	Definition of Controls	Comparability of Cases and Controls on the Basis of the Design or Analysis	Ascertainment of Exposure	Same Method of Ascertainment for Cases and Controls	Non-Response Rate	Total Score
Barbier et al. (2011) [25]	1	1	0	1	1	1	1	1	7
Fujii et al. (2017) [28]	1	1	0	1	2	1	1	0	7
Mokdad et al. (2017) [30]	1	1	0	1	2	1	1	0	7
Papalezova et al. (2012) [31]	1	1	0	1	2	1	1	1	8
Roland et al. (2015) [33]	1	1	0	1	1	1	1	0	6
Sho et al. (2015) [35]	1	1	0	1	2	1	1	0	7
Tzeng et al. (2014) [36]	1	1	0	1	2	1	1	1	8
Vidri et al. (2021) [37]	1	1	0	1	2	1	1	1	8
Yoon et al. (2022) [38]	1	1	0	1	2	1	1	1	8

A score of less than 4 points indicates a high risk of bias; a score of 5 indicates a moderate risk of bias; and a score of more than 5 indicates a low risk of bias.

**Table 2:** Study Characteristics.

Author	Country	Period	Multicenter Study	Study Design	Sample			Median OS
					Total	NAT	US	
Barbier et al. (2011) [25]	France	1997–2006	No	Retrospective Study	173	88	85	NAT: 15 mo (3–72)
								US: 17 mo (1–109)
Birrer et al. (2021) [26]	Switzerland	2009–2018	No	RCT	34	16	18	NR
Casadei et al. (2015) [27]	Italy	2007–2014	No	RCT	38	18	20	NAT: 22.4 mo (10.2–34.6)
								US: 19.5 mo (7.5–31.5)
Fujii et al. (2017) [28]	Japan	2001–2013	Yes	NR	273	40	233	NAT: 24.9 mo
								US: 23.5 mo
Golcher et al. (2015) [29]	Germany	2003–2009	Yes	RCT	66	33	33	NAT: 17.4 mo
								US: 17.4 mo
Mokdad et al. (2017) [30]	USA	2006–2012	No	NR	8020	2005	6015	NAT: 26 mo
								US: 21 mo
Papalezova et al. (2012) [31]	USA	1999–2007	No	Retrospective Study	236	144	92	NAT: 15 mo
								US: 13 mo
Reni et al. (2018) [32]	Italy	2010–2015	No	RCT	62	32	30	NAT: 38.2 mo (27.3–49.1)
								US: 26.4 mo (15.8–26.7)
Roland et al. (2015) [33]	USA	1990–2008	No	Retrospective Study	307	222	85	NR
								NR
Seufferlein et al. (2023) [34]	Germany	2015–2019	Yes	RCT	118	59	59	NAT: 25.5 mo (19.7–29.7)
								US: 16.7 mo (11.6–22.2)
Sho et al. (2015) [35]	Japan	2006–2013	No	Retrospective Study	100	44	56	NAT: 50.2 mo
								US: 32.7 mo
Tzeng et al. (2014) [36]	USA	2002–2007	No	Retrospective Study	167	115	52	NAT: 28 mo (21.7–34.3)
								US: 25.3 mo (19.9–30.7)
Versteijne et al. (2022) [18]	Netherlands	2013–2017	Yes	RCT	133	65	68	NAT: 15.7 mo (12.9–20.6)
								US: 14.3 mo (12.7–17.9)
Vidri et al. (2021) [37]	USA	1989–2021	Yes	Retrospective Study	6802	1118	5684	NAT: 27.6 mo (IQR: 38.8)
								US: 25.6 mo (IQR: 40.9)
Yoon et al. (2022) [38]	Korea	2012–2019	No	Retrospective Study	202	167	35	NR
								NR

NAT: Neoadjuvant Therapy; US: Upfront Surgery; NR: Not reported; RCT: Randomized Controlled Trial.

## Supplementary Materials

The following supporting information can be downloaded at: https://www.mdpi.com/article/10.3390/cancers15184627/s1, Figure S1: Overall Survival: Galbraith Plot; Figure S2: Overall Survival: Subgroup analysis related to study design—(RCT = 6, retrospective studies = 7, Study design Not Reported = 2); Figure S3: Overall Survival: Cumulative analysis per year; Figure S4: Overall Survival: Leave-one-out analysis; Figure S5: Overall Survival: Funnel plot for publication bias; Figure S6: Disease Free-Survival: Galbraith Plot; Figure S7: Disease Free Survival (n = 7): Forest plot—Subgroup analysis related to use of neoadjuvant chemotherapy or neoadjuvant chemoradiotherapy; Figure S8: Disease Free Survival—Subgroup analysis related to study design (RCT = 6, retrospective studies = 1); Figure S9: Disease Free Survival: Cumulative Meta-Analysis; Figure S10: Disease Free Survival: Leave-one-out analysis; Figure S11: R0 Rate: L’Abbè Plot for R0 Rate Studies; Figure S12: R0 Rate—Subgroup analysis related to study design (RCT = 5, retrospective studies = 6, Study design Not Reported = 2); Figure S13: R0 Rate (n = 13): Cumulative Meta-Analysis; Figure S14: R0 Rate (n = 13): Leave-one-out Analysis; Figure S15: R0 Rate (n = 13): Funnel Plot—Publication Bias; Figure S16: N+ Rate (n = 8): L’Abbé Plot—N+ Rate; Figure S17: N+ Rate (n = 8): Sensitive Analysis; Figure S18: N+ Rate (n = 8): Cumulative meta-analysis; Figure S19: N+ Rate—Subgroup analysis related to study design (RCT = 2, retrospective studies = 5, Study design Not Reported = 1); Table S1: Studies Characteristics;
