# Supplementary material for: Neo-Adjuvant Treatment in Primary Resectable Pancreatic Cancer: A Systematic Review and PRISMA-Compliant Updated Metanalysis of Oncological Outcomes
Source: Cancers (Basel). 2023 Sep 19;15(18):4627. doi: 10.3390/cancers15184627 (PMC10526896; doi:10.3390/cancers15184627)
Supplement: Supplementary file 1 [file cancers-15-04627-s001.zip › cancers-2627078-supplementary.pdf]

Table S1. Studies Characteristics.

Author	Neoadjuvant Regimen	Adjuvant Chemotherapy	Gender (Male/Female)	Age *
Barbier et al. (2011)	5 FU + Cisplatin + RT	None	NR	NAT: 65 (39-81)
	Upfront Surgery	5 FU or Gem		US: 64 (37-79)
Birrer et al. (2021)	Gem + Oxaliplatin	Gem	NR	NR
	Upfront Surgery	Gem		
Casadei et al. (2015)	Gem 6 weeks + (Gem+RT) 6 weeks	Gem	NAT: 8/10	NAT: 71.5 (51-78)
	Upfront Surgery	Gem	US: 14/6	US: 67.5 (48-79)
Fujii et al. (2017)	5 FU + Oteracil and Gimeracil + S-1 RT	Gem or Capecitabine	NAT: 21/19	NAT: 65 (36-79)
	Upfront Surgery	Gem + Capecitabine	US: 146/87	US: 67 (35-88)
Golcher et al. (2015)	Gem + Cispatin + RT	Gem	NAT: 17/16	NAT: 62.5 (33-76)
	Upfront Surgery	Gem	US: 18/15	US: 65.1 (46-73)
Mokdad et al. (2017)	Chemotherapy (NR)	-	NAT: 1044/961	NAT: 18-55: 340, 55-64: 650; 65-74: 729; ≥ 75: 286;
	Upfront Surgery	-	US: 3150/2865	US: 18-55: 985; 55-64: 1972; 65-74: 2222; ≥ 75: 836
Papalezova et al. (2012)	5-FU + RT	Gem	NAT: 75/69	NAT: 64 ± 12
	Upfront Surgery	5FU	US: 49/43	US: 65 ± 12
Reni et al. (2018)	Capecitabine + Gem + Cisplatin + Epirubicin	Capecitabine + Gem + Cisplatin + Epirubicin	NAT: 25/7	NAT: 64 (39-75)
	Upfront Surgery	Capecitabine + Gem + Cisplatin + Epirubicin	US: 13/17	US: 68 (49-75)
Roland et al. (2015)	Gem of 5 FU + RT	Gem (10%)	NAT: 126/96	NAT: 64 (35-86)
	Upfront Surgery	Gem ± RT	US: 43/42	US: 64 (40-85)
Seufferlein et al. (2023)	Gem + Abraxane		NAT: 34/25	NAT: 65 (48-82);
	Upfront Surgery	Gem	US: 37/22	US: 68 (41-88)
Sho et al. (2015)	Gem + RT or Capecitabine	WHF + Gem or Gem alone	NAT: 24/20	NAT: 66.4 ± 8.5
	Upfront Surgery	WHF + Gem	US: 32/24	US: 69.7 ± 9.1
Tzeng et al. (2014)	Gem + Cis + RT	-	NAT: 61/54	NAT: 65.5 (38-79)
	Upfront Surgery	Gem	US: 30/22	US: 61.9 (25-79)
Verstaijne et al. (2022)	Gem + RT	Gem	NAT: 64/55	NAT: 66 (59.71)
	Upfront Surgery	Gem	US: 74/53	US: 67 (60-73)
Vidri et al. (2021)	Chemotherapy (NR)	NR	NAT: 600/518;	NAT: 63.4 (10.0);
	Upfront surgery		US: 1304/1502	US: 69.0 (10.9)
Yoon et al. (2022)	Gem + 5-FU + Leucovorin, Irinotecan + Oxaplatin	Gem + 5-FU + Leucovorin, Irinotecan + Oxaplatin	NAT: 21/14;	NAT: 67.0 (58-73);
	Upfront Surgery	Gem + 5-FU + Leucovorin, Irinotecan + Oxaplatin	US: 73/94	US: 60 (56-70)

* Age is reported as median (range) or mean ± standard deviation – Gem: gemcitabine; 5-FU: 5-fluorouracil; NAT: Neoadjuvant Therapy; US: US – NR: Not reported

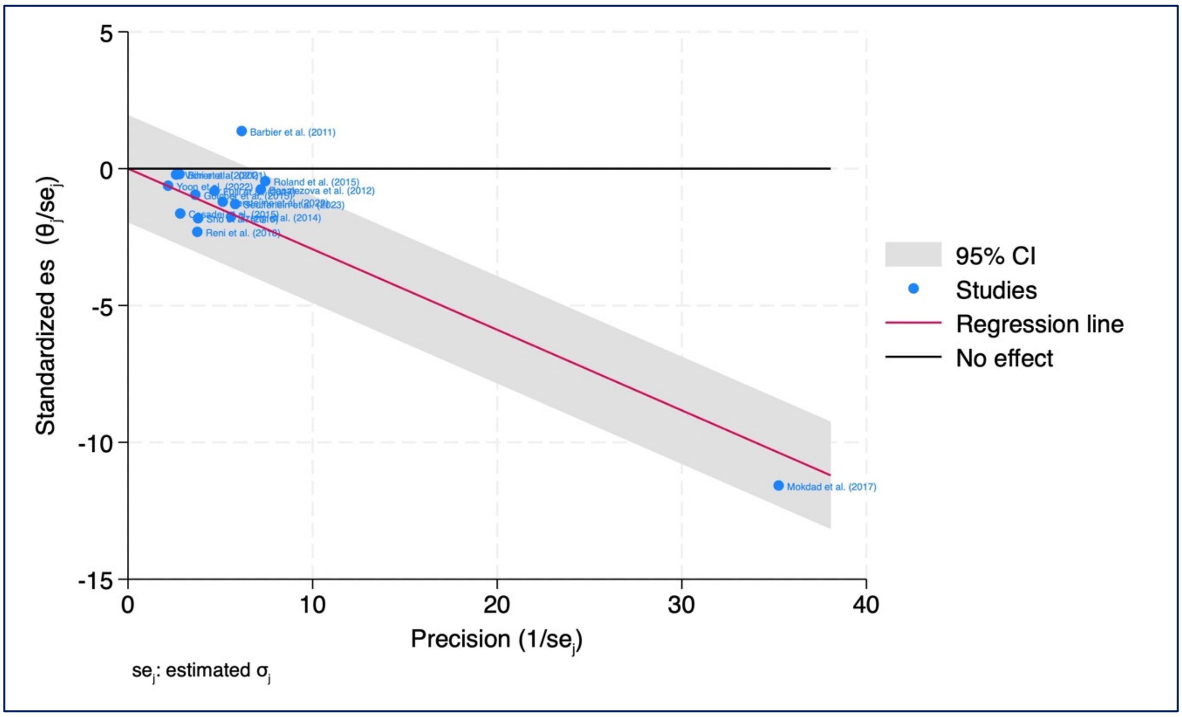

Figure S1. Overall Survival: Galbraith Plot.

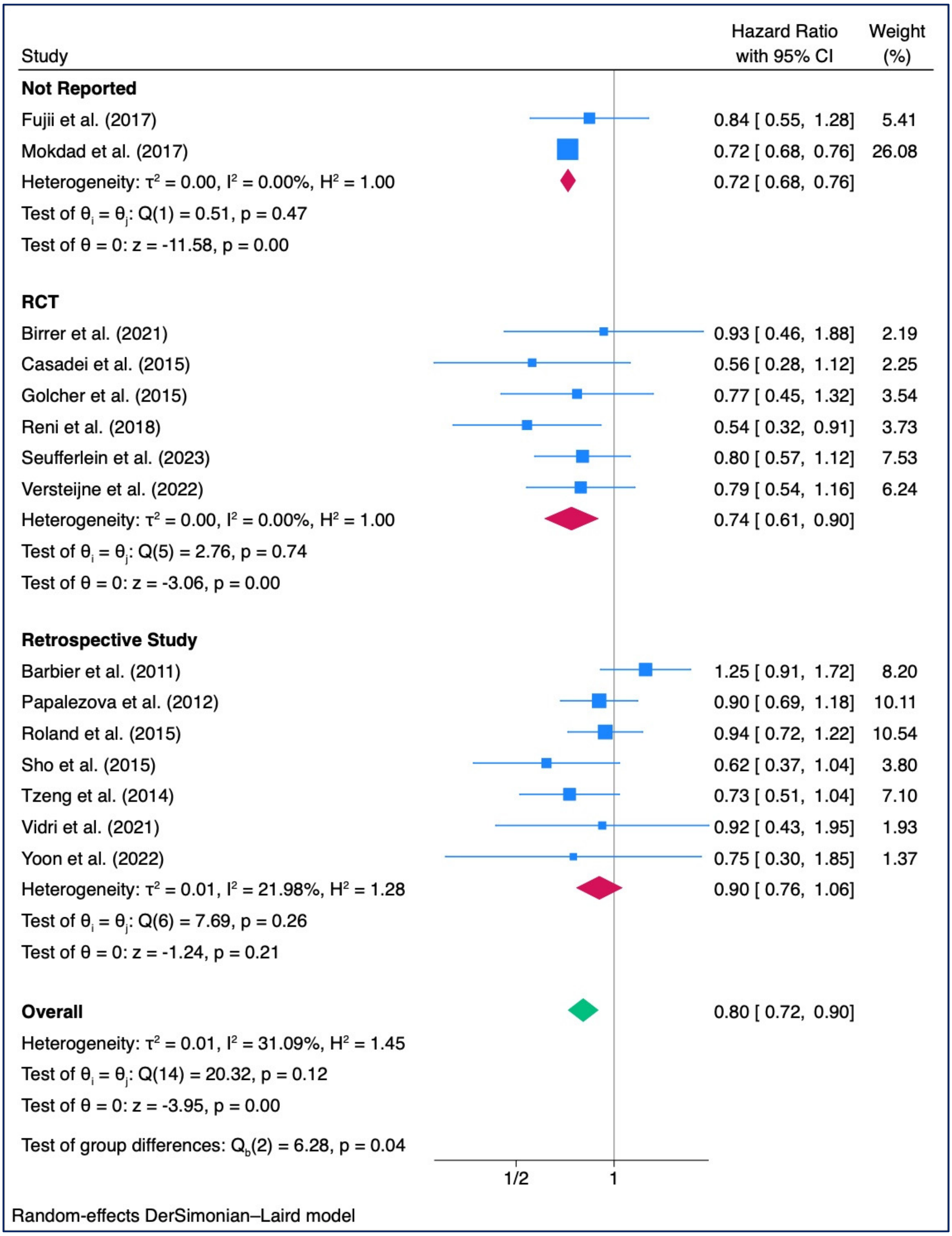

Figure S2. Overall Survival: Subgroup analysis related to study design—(RCT = 6, retrospective studies = 7, Study design Not Reported = 2).

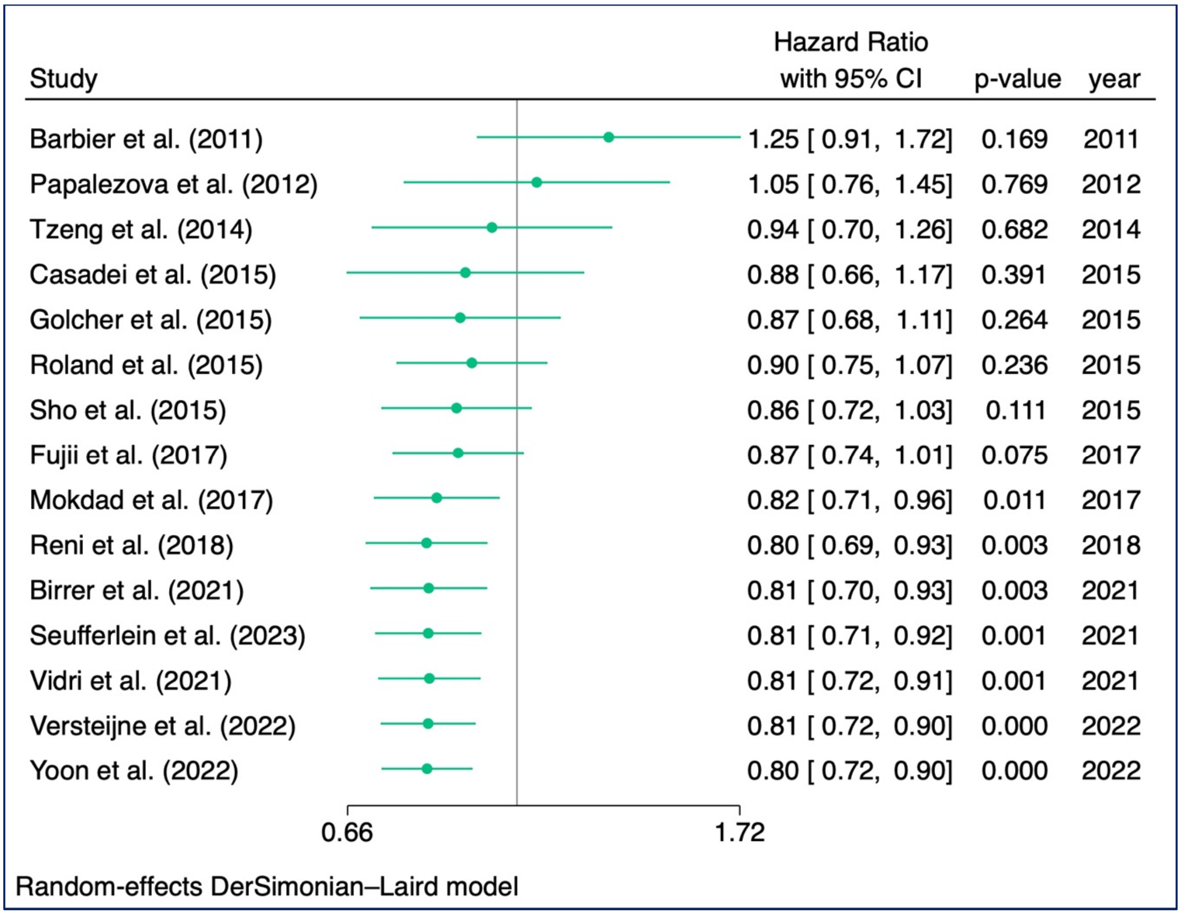

Figure S3. Overall Survival: Cumulative analysis per year.

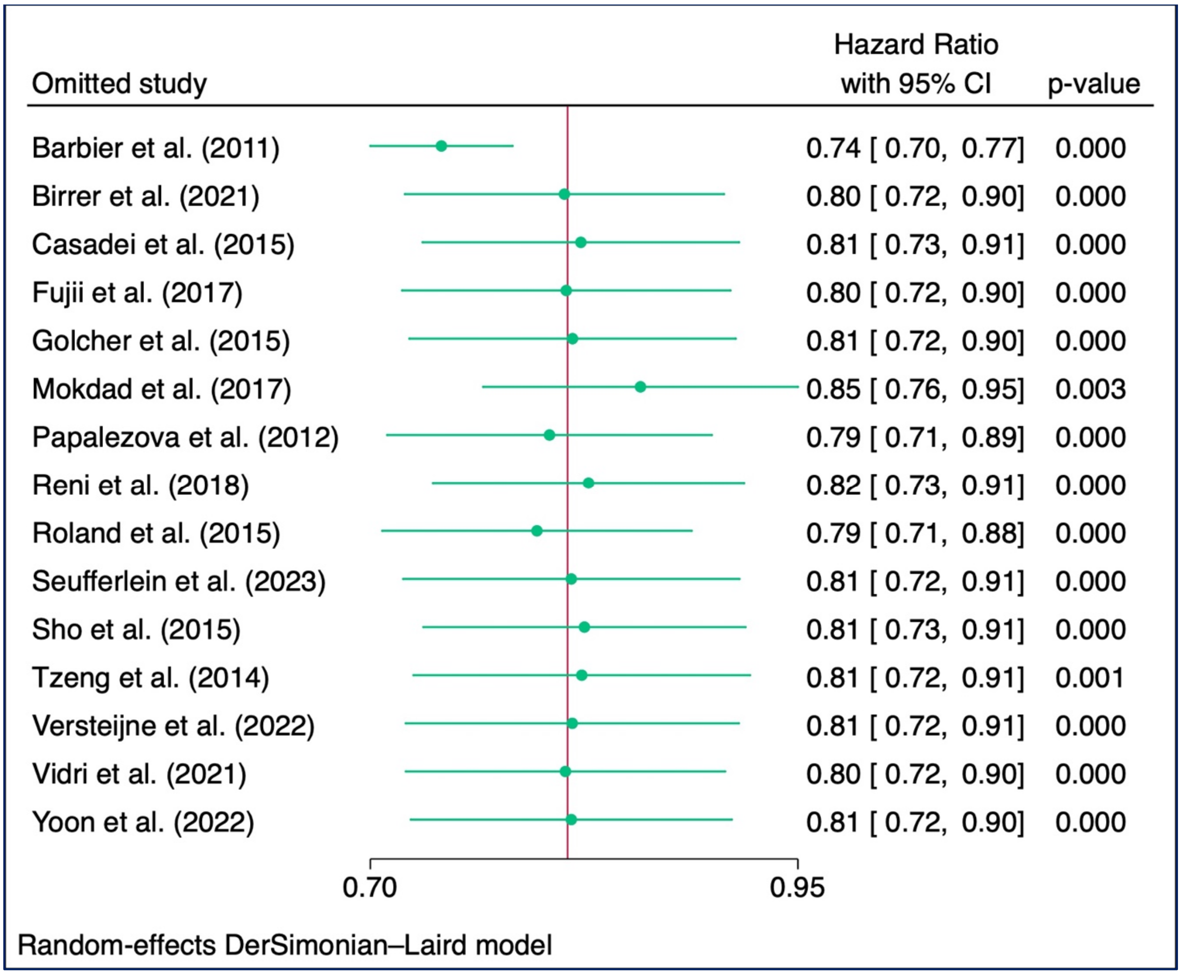

Figure S4. Overall Survival: Leave-one-out analysis.

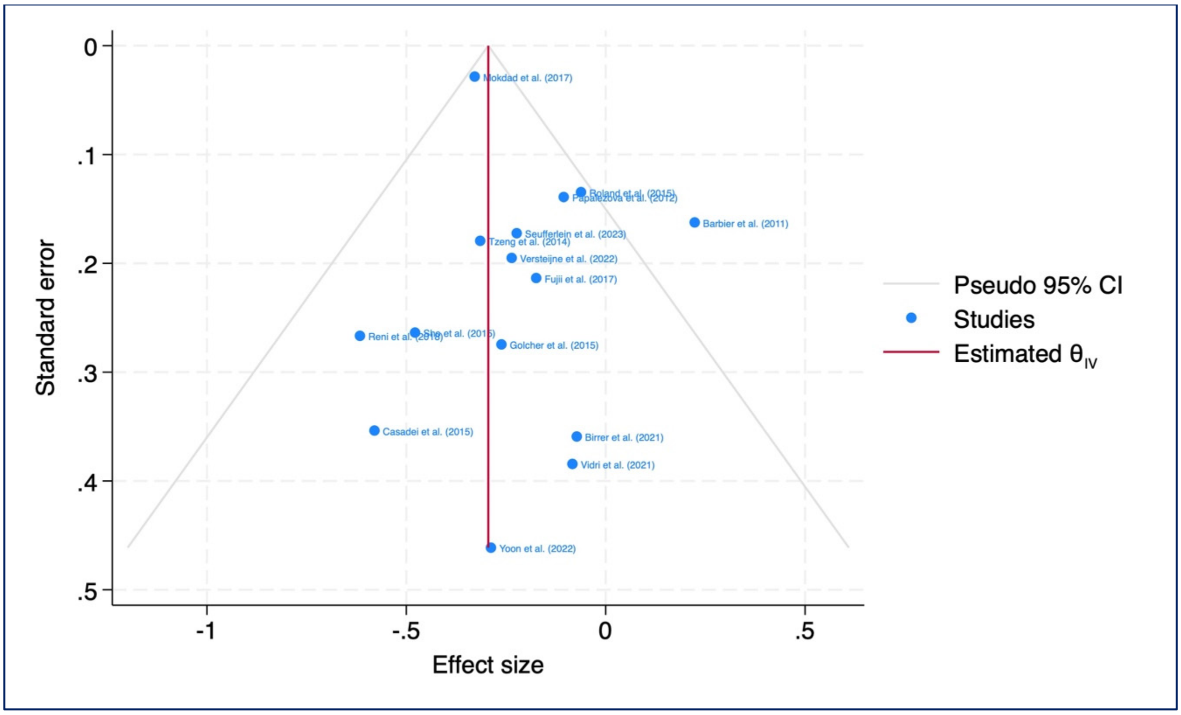

Figure S5. Overall Survival: Funnel plot for publication bias.

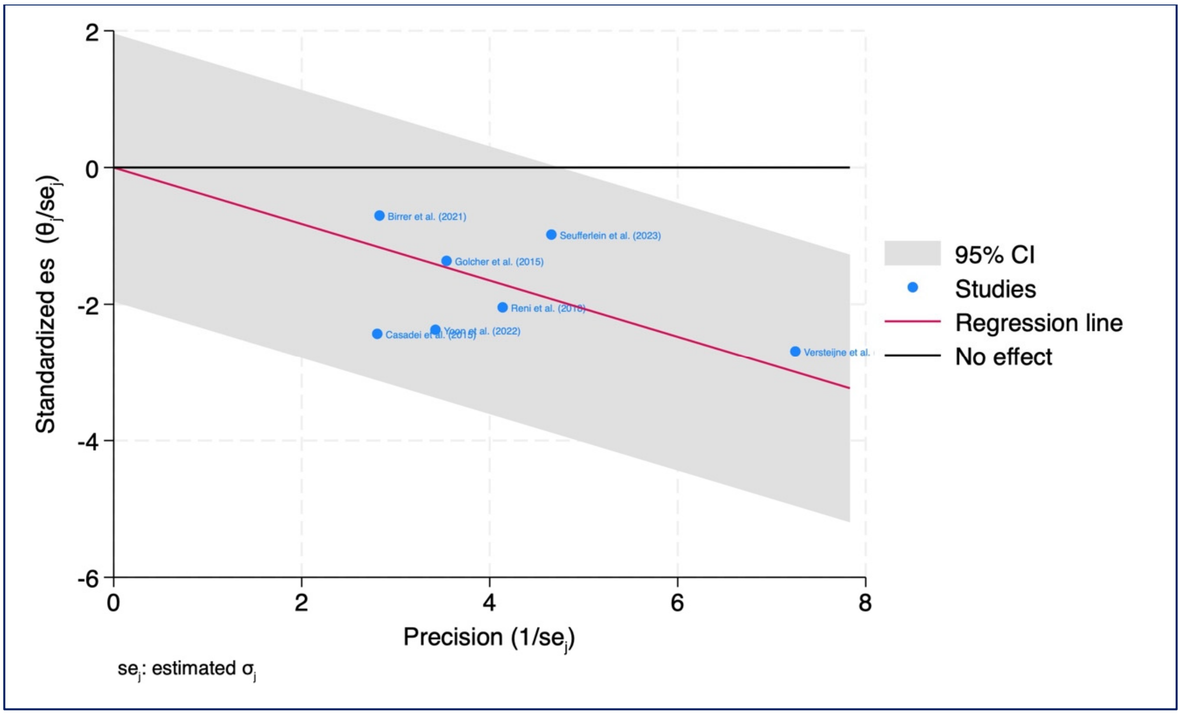

Figure S6. Disease Free-Survival: Galbraith Plot.

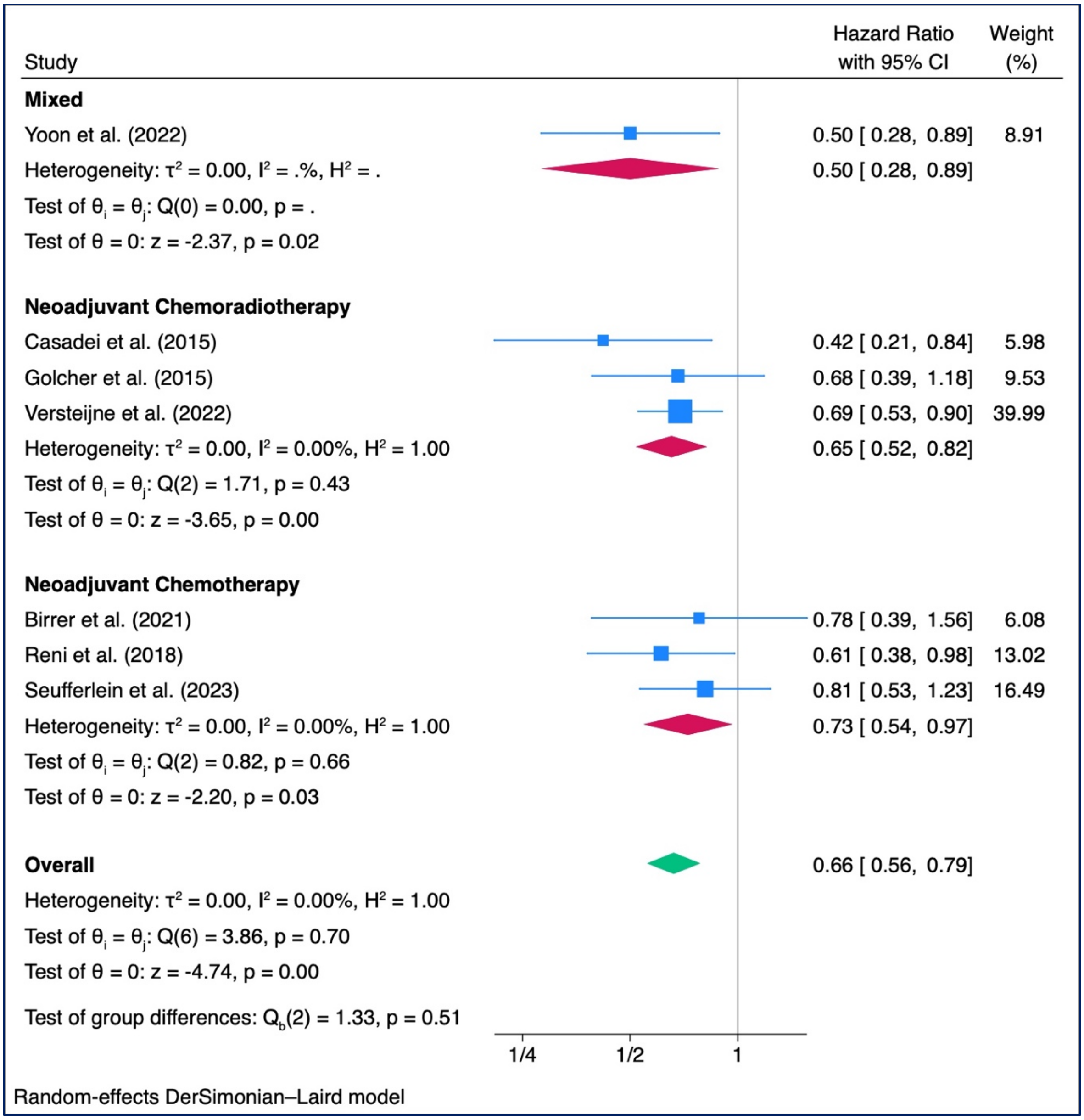

Figure S7. Disease Free Survival (n = 7): Forest plot—Subgroup analysis related to use of neoadjuvant chemotherapy or neoadjuvant chemoradiotherapy.

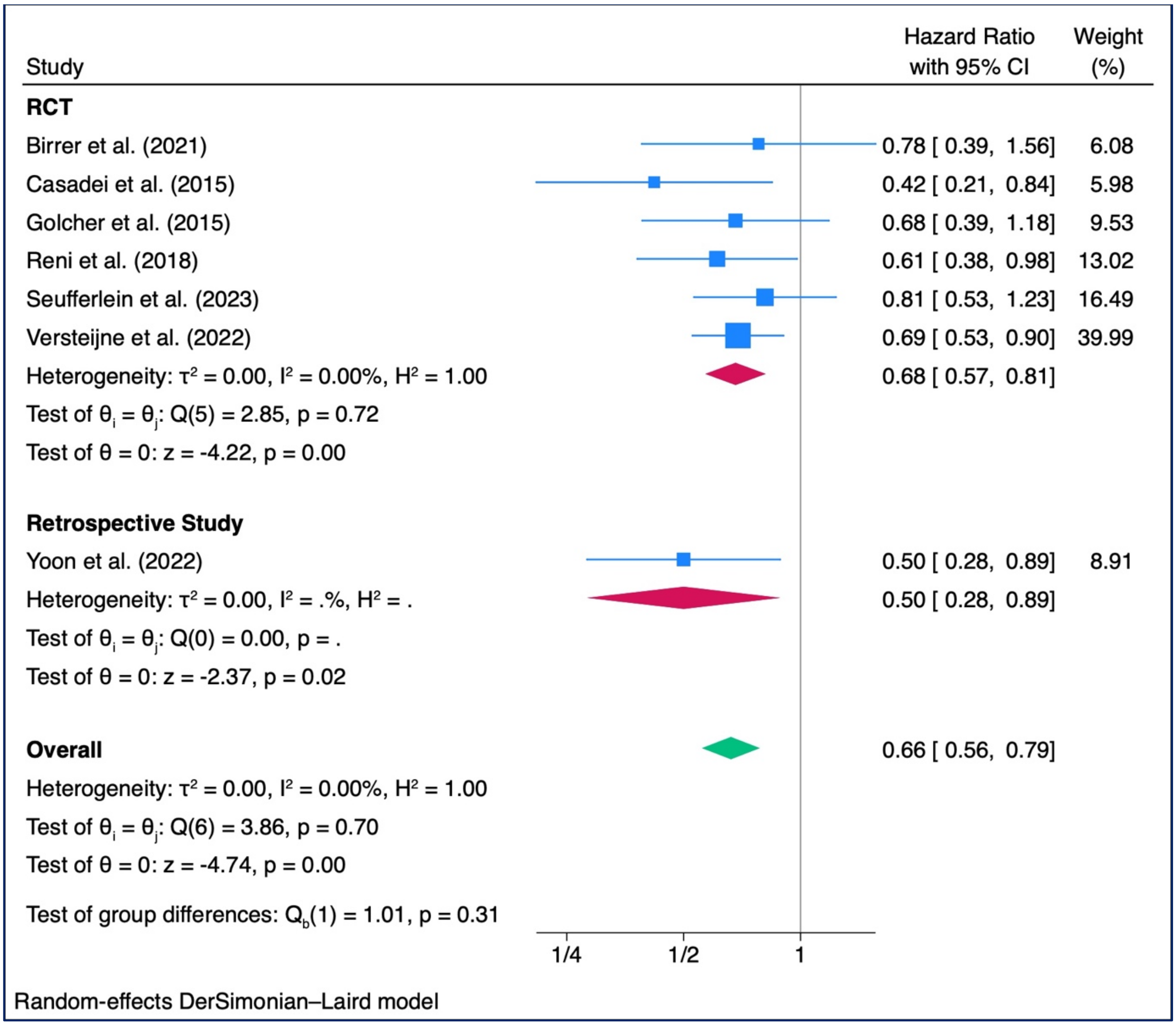

Figure S8. Disease Free Survival—Subgroup analysis related to study design (RCT = 6, retrospective studies = 1).

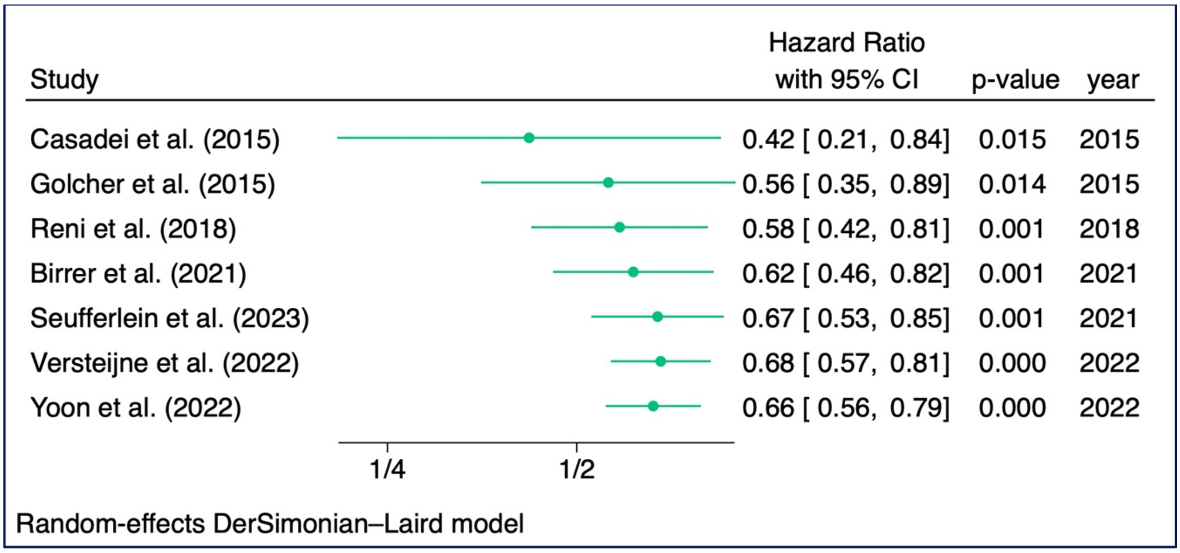

Figure S9. Disease Free Survival: Cumulative Meta-Analysis.

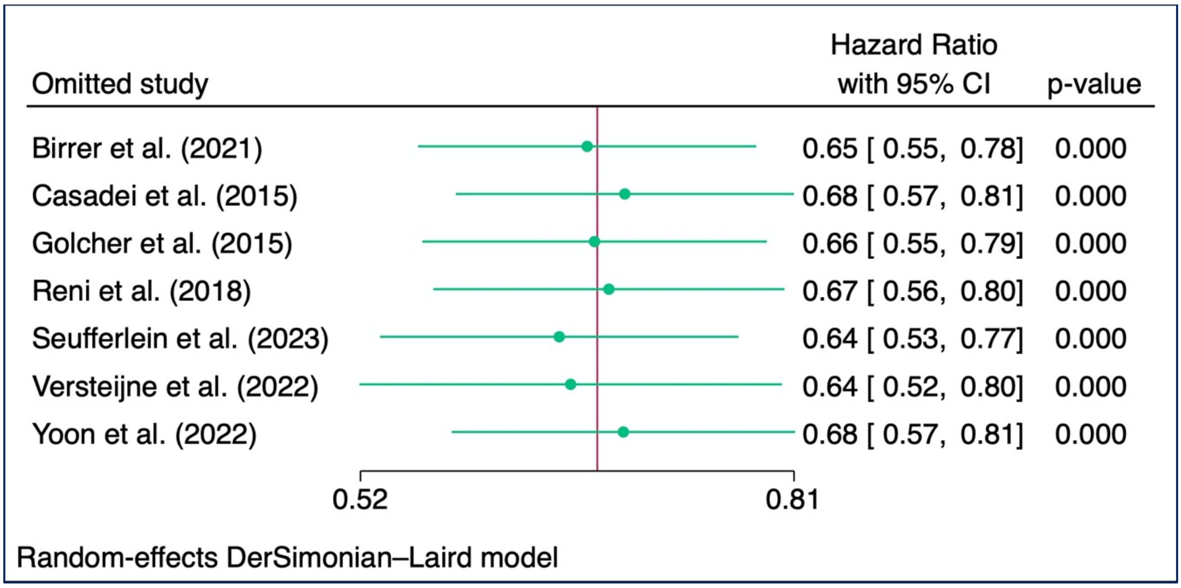

Figure S10. Disease Free Survival: Leave-one-out analysis.

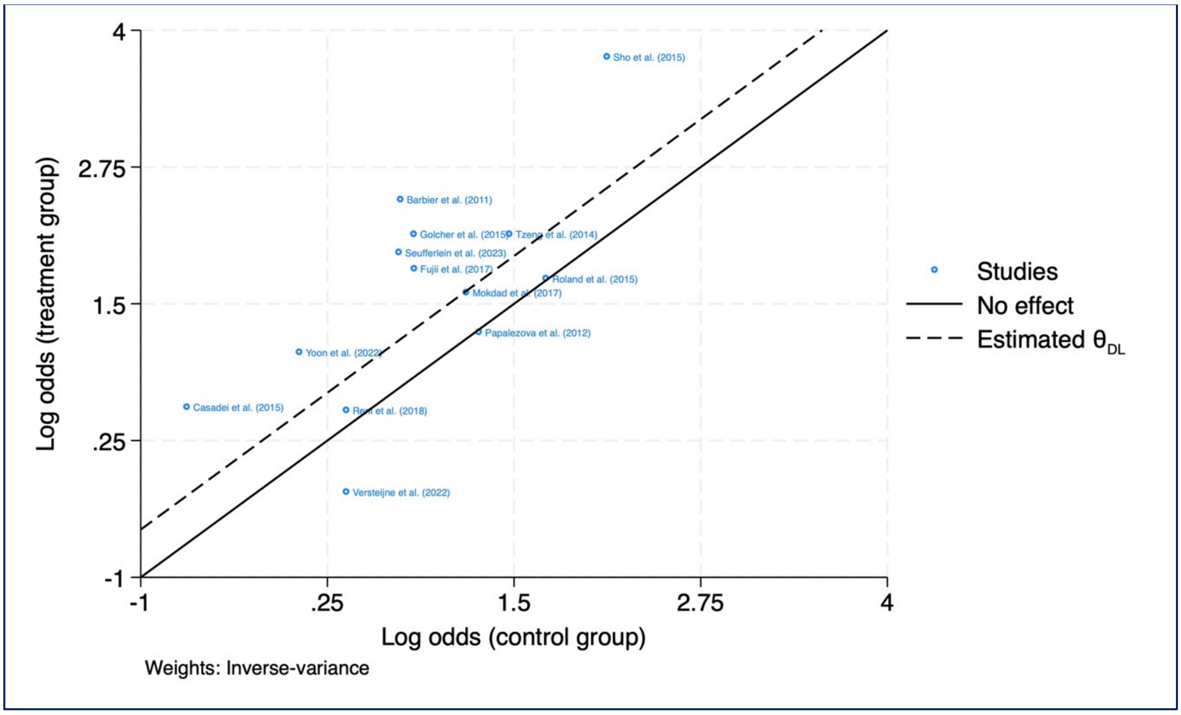

Figure S11. R0 Rate: L′Abbè Plot for R0 Rate Studies.

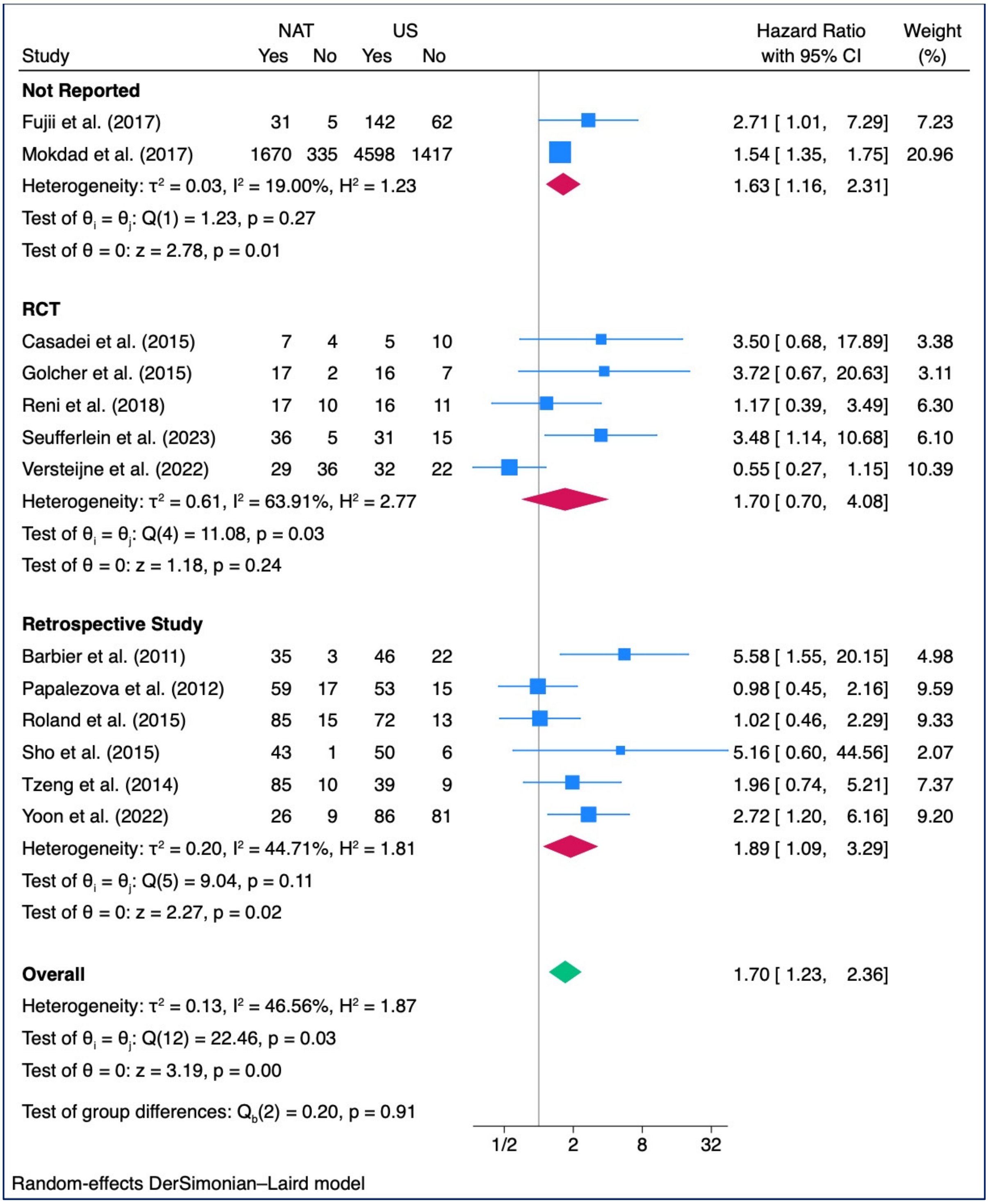

Figure S12. R0 Rate—Subgroup analysis related to study design (RCT = 5, retrospective studies = 6, Study design Not Reported = 2).

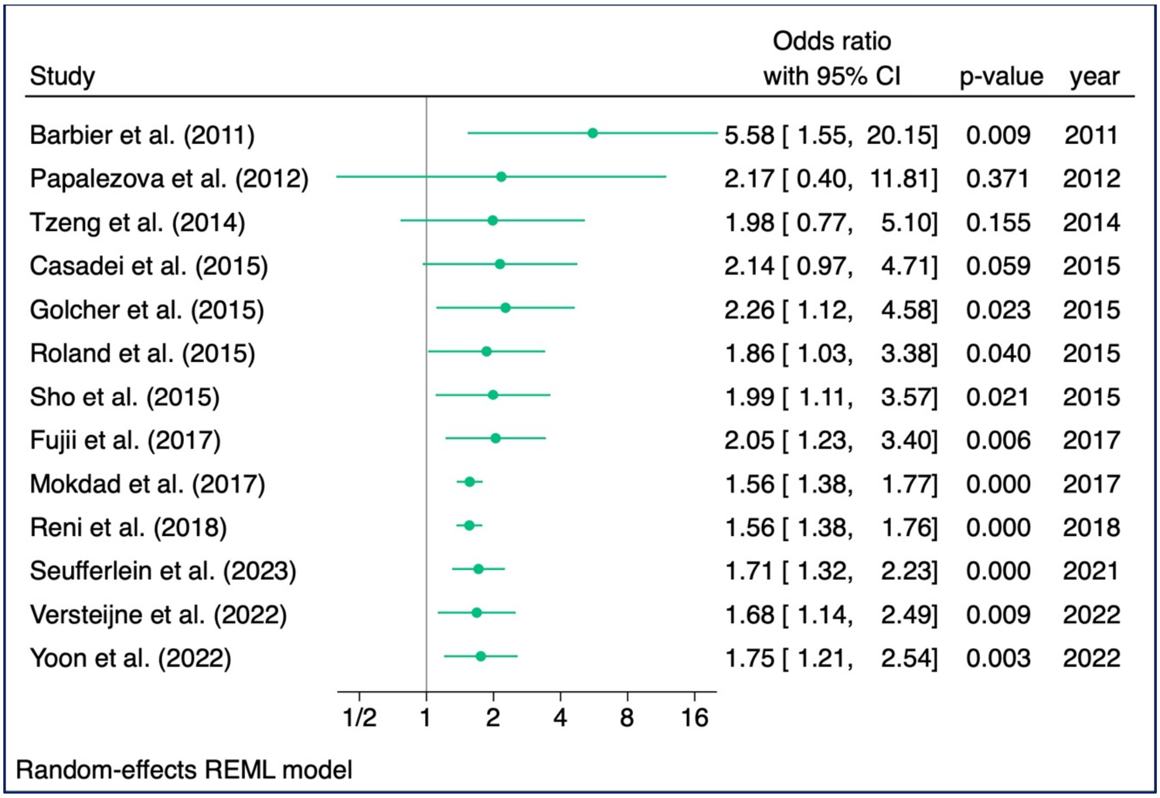

Figure S13. R0 Rate (n = 13): Cumulative Meta-Analysis.

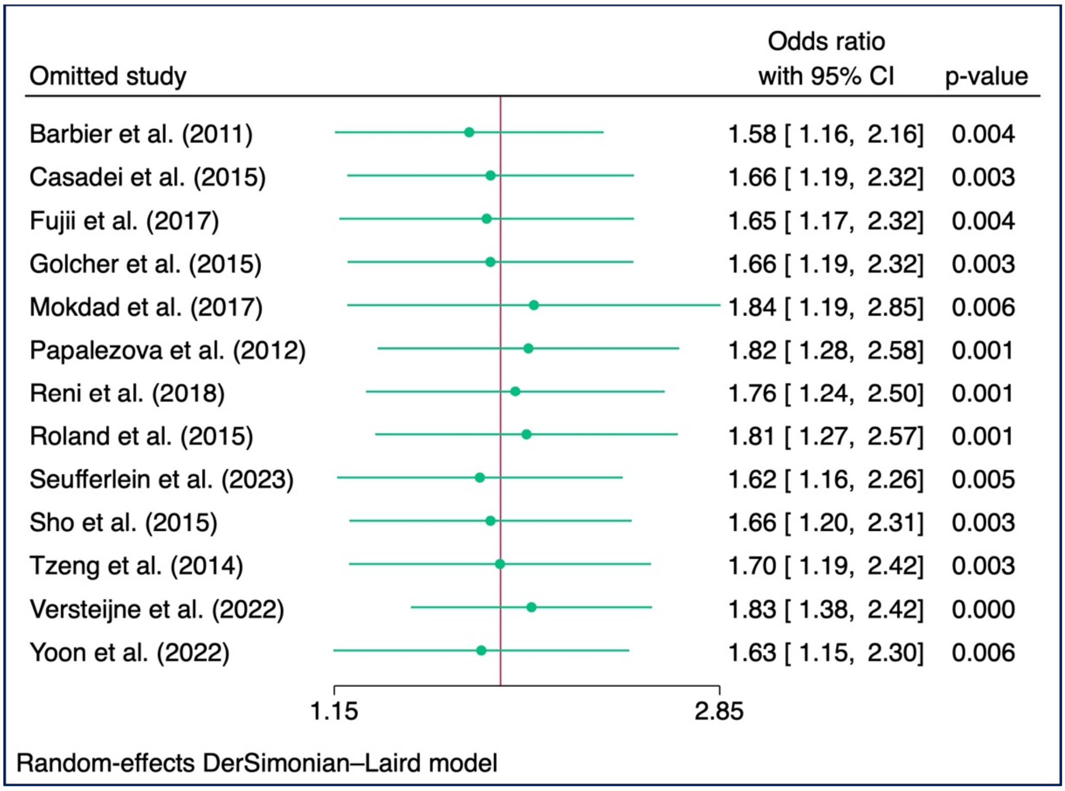

Figure S14. R0 Rate (n = 13): Leave-one-out Analysis.

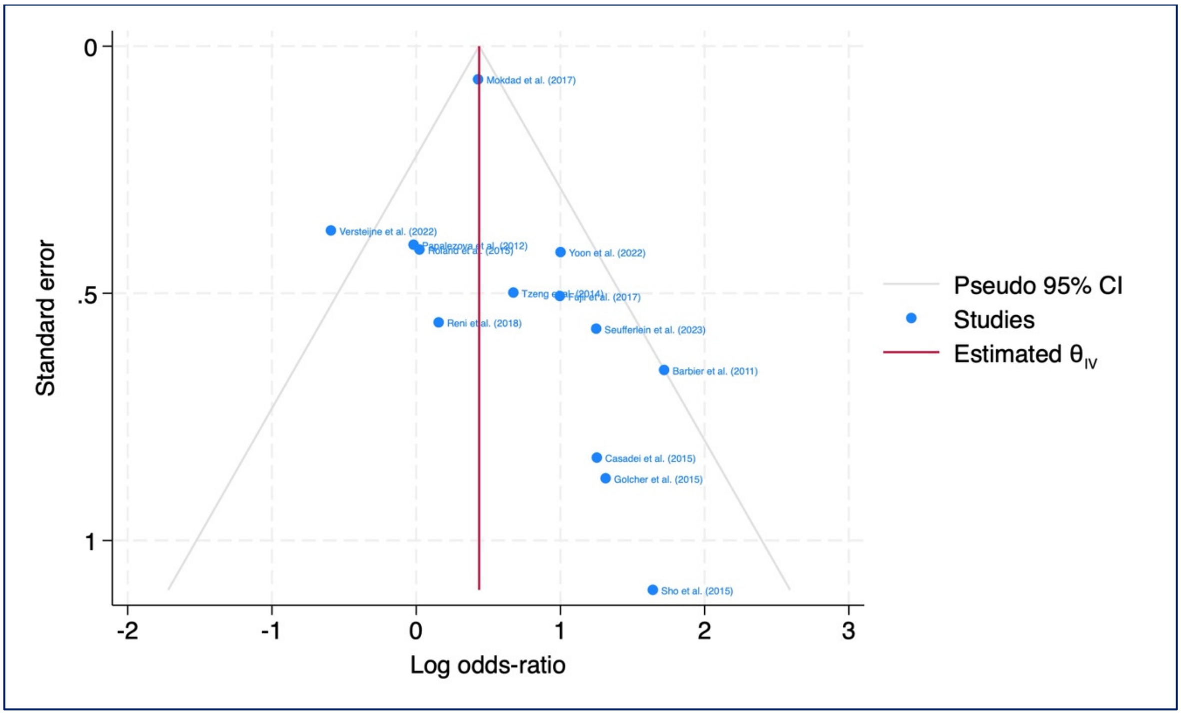

Figure S15. R0 Rate (n = 13): Funnel Plot—Publication Bias.

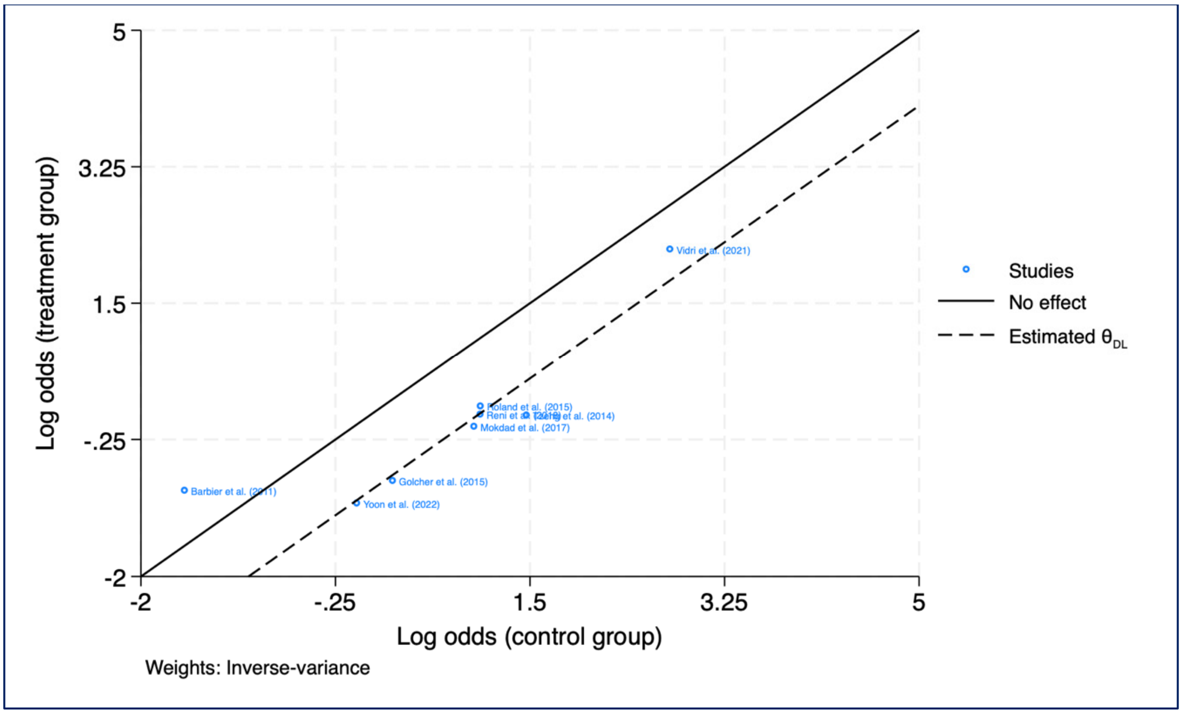

Figure S16. N+ Rate (n = 8): L'Abbé Plot—N+ Rate. Note: for Verstejine et al. 2022 data were extracted from the Verstejine et al. 2020.

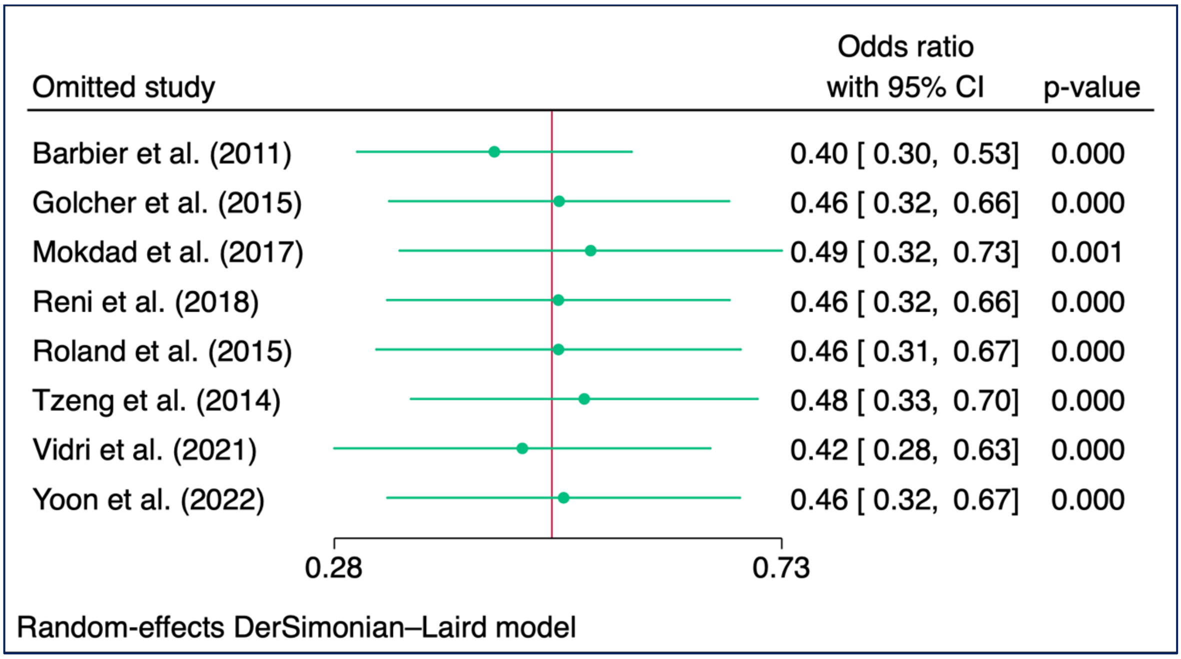

Figure S17. N+ Rate (n = 8): Sensitive Analysis.

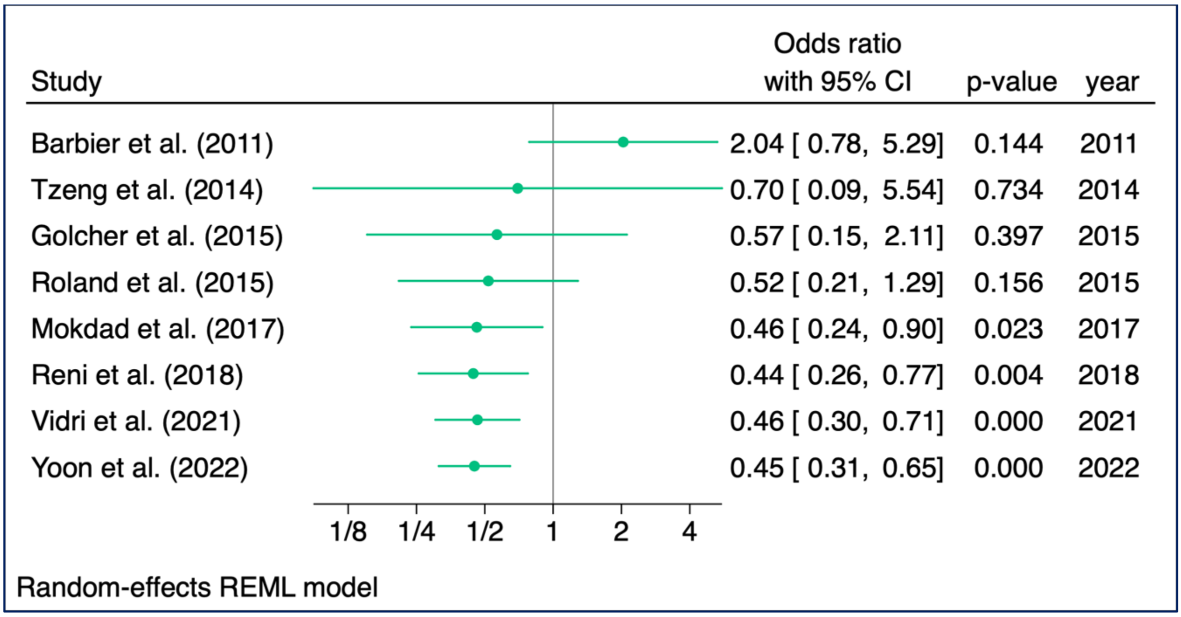

Figure S18. N+ Rate (n = 8): Cumulative meta-analysis.

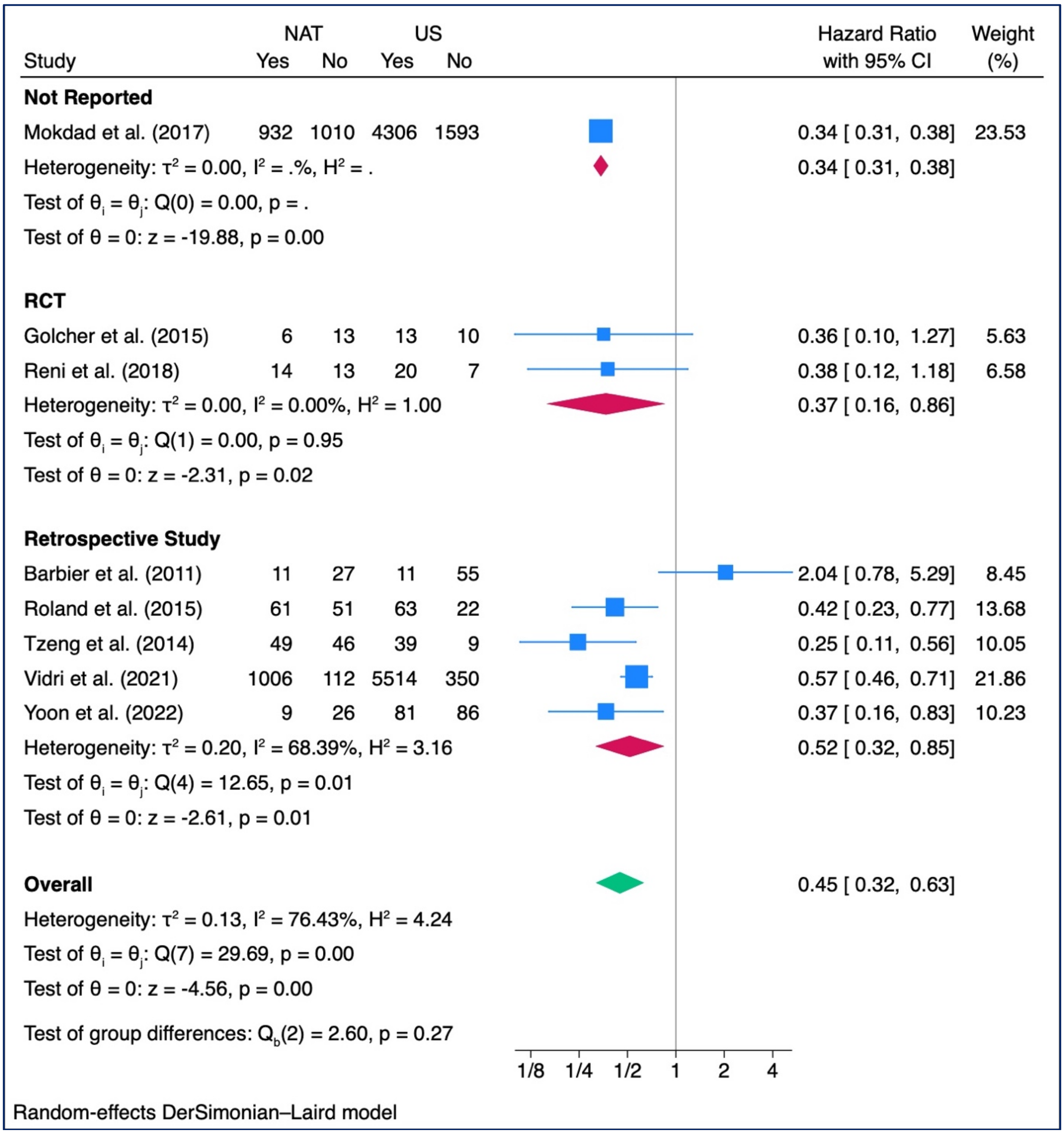

Figure S19. N+ Rate—Subgroup analysis related to study design (RCT = 2, retrospective studies = 5, Study design Not Reported = 1).
